# Soluble sugars make a greater contribution than cell wall components to the variability of freezing tolerance in wheat cultivars

**DOI:** 10.5511/plantbiotechnology.24.0801a

**Published:** 2024-12-25

**Authors:** Sushan Chowhan, Takuma Kikuchi, Momoka Ohashi, Tatsuya Kutsuno, Hiroto Handa, Toshihisa Kotake, Daisuke Takahashi

**Affiliations:** 1 Graduate School of Science and Engineering, Saitama University, Saitama 338-8570, Japan; 2 Bangladesh Institute of Nuclear Agriculture, Sub-station Ishurdi, Pabna-6620, Bangladesh

**Keywords:** cell wall, cold acclimation, freezing tolerance, soluble sugars, wheat

## Abstract

Wheat, the second most produced cereal globally, is primarily cultivated in cooler regions. Unexpected freezing temperatures can severely impact wheat production. Wheat and other temperate plants have a cold acclimation mechanism that enhances freezing tolerance, but reduces growth under low, non-freezing temperatures. During cold acclimation, plants break down storage polysaccharides like starch and fructan to accumulate soluble sugars such as glucose and fructose. These soluble sugars aid freezing tolerance through osmotic adjustments, membrane stabilization, and freezing point depression. However, plant cell walls, composed of insoluble polysaccharides, are the first line of defense against extracellular freezing. We analyzed the contributions of soluble sugars, storage polysaccharides, and cell wall polysaccharides to freezing tolerance and growth under cold acclimation in wheat. The study involved two Japanese winter cultivars (Yumechikara and Norin-61) and one Japanese spring cultivar (Haruyokoi). While Yumechikara showed poor growth after four weeks of cold acclimation, it exhibited higher freezing tolerance than the other cultivars. Our analysis revealed that Yumechikara accumulated higher levels of glucose, fructose, starch, and fructan than Norin-61 and Haruyokoi, whereas no significant differences in cell wall composition among the cultivars were observed. Gene expression patterns related to soluble sugar metabolism supported these findings. Additionally, the distribution of sugar changes between leaves (source) and crown (sink) correlated with the relationship between growth and freezing tolerance. These results suggest that freezing tolerance in wheat involves a balance between sugar accumulation and growth regulation during cold acclimation.

## Introduction

Growth and development of plants are significantly influenced by freezing or low positive temperatures. Two thirds of the earth’s surface experience sub-zero temperature annually (Rihan et al. 2017). In temperate regions, the successful establishment of crops is contingent upon adequate cold hardiness, achieved through tolerance or avoidance of freezing in plant tissues (Willick et al. 2021). Wheat is the second most produced cereal after maize (FAOSTAT 2024) and tolerant to freezing (Thomashow 1998), which makes it different from the other major cereals maize and rice which are susceptible to cold stress (Hu et al. 2017; Kim and Tai 2011). However, severe low temperatures during winter are a major constraint in wheat production (Jing-Song et al. 2012). In fact, regions such as China (Xiao et al. 2018), the USA (Holman et al. 2011), Europe (Trnka et al. 2014), Australia (Crimp et al. 2016) and Canada (Willick et al. 2021) face unexpected temperature changes, particularly severe low temperatures after winter thaws due to global warming and climate change.

The process leading to freezing injury begins when ice is initially formed in the large intercellular spaces (extracellular region) and spreads from nucleation points, resulting in the formation of ice crystals. Under severe low temperatures, ice crystals in the apoplastic region create a difference in the chemical potential between the inside and outside of the cell, driving the movement of water from the cell to the extracellular space and compressing the cell wall (Pearce 2001).

Moreover, extracellular freezing at lower subzero temperatures leads to severe deformation of the cells via cytorrhysis due to the growth of extracellular ice crystals and severe dehydration. Eventually, this causes irreversible deformation of the plasma membrane ultrastructure through unfavorable interaction with endomembranes, leading to cell death (Fujikawa 2016; Gusta et al. 1975; Levitt 1980; Mazur 1963). Thus, freezing injury is a complex mechanism.

Prolonged low temperature treatment reduces plant growth rate compared to untreated plants, resulting in reduced leaf size, leaf area, shoot biomass, and effects on root growth (Buriro et al. 2011; Kul et al. 2020; Valluru et al. 2012). However, when temperate plants are exposed to low and non-freezing temperatures, they undergo an adaptation process to enhance freezing tolerance, known as cold acclimation (CA), although growth rate is suppressed. In the CA period, arrested growth is accompanied by various physiological events such as the accumulation of soluble sugars that prepare the plant for further cold condition (Thomashow 1999).

Winter wheat cultivars can continue carbon assimilation even at cold temperatures, but spring cultivars cannot (Savitch et al. 1997). Autumn-seeded wheat cultivars (winter wheat, e.g. Norstar) can endure temperatures as low as −20°C after CA, whereas spring wheat cultivars, devoid of a vernalization or daylength requirement and spending less time in the vegetative phase, exhibit reduced CA development and freezing tolerance (Mahfoozi et al. 2006). In fact, the 50% lethal temperature (LT_50_) of the Iranian spring wheat cultivar Kohdasht was found to be −6°C at its highest freezing tolerance, even after CA, while before CA, LT_50_ in Norstar and Kohdasht are not dramatically different (−8°C and −3°C, respectively). Therefore, CA is an important mechanism to improve the freezing tolerance in wheat. However, in wheat, the crown is the most important tissue as it includes meristematic tissues and ultimately determines the ability to regrow after severe temperature conditions. The crown part includes the shoot apical meristem consisting of leaf meristem tissues and developing leaf buds, whereas the vascular transition zone comprises xylem, pith, and root meristematic tissues (Willick et al. 2018).

One important cellular change in the CA process is the accumulation of soluble sugars to regulate osmotic pressure, stabilize membranes and attain freezing point depression (Koster and Lynch 1992). The accumulation of soluble sugars can be primarily achieved by storage polysaccharides such as starch and sucrose (Suc), thus the degradation of these by enzyme action is also important in the study of the mechanism of soluble sugar accumulation or degradation in plants. It is well known that invertase and α-amylase are key enzymes involved in the degradation of Suc and starch, which yield reducing sugars like glucose (Glc), and fructose (Fru). Accordingly, a differential level of gene expression is observed in response to cold stress. In wheat, a certain number of genes that are responsible for the synthesis and degradation of Suc (*TaINV*, *Triticum aestivum Invertase*; *TaSUS1*, *T. aestivum Suc synthase 1*; *TaSPS*, *T. aestivum Suc phosphate synthase*) (Bagherikia et al. 2018) and starch (*TaAMY1*, *T. aestivum amylase 1*; *TaSS1*, *T. aestivum starch synthase 1*; *TaAGPS*, *T. aestivum ADP-Glc pyrophosphorylase small subunit*; *TaAGPL*, *T. aestivum ADP-Glc pyrophosphorylase large subunit*) (Barrero et al. 2013; Gu et al. 2021; Wang et al. 2014; Zhang et al. 2019) have been studied and found crucial for sugar metabolism during CA. In plants of the Poaceae family, fructan, that is, storage Fru oligosaccharides and polysaccharides, have also been found to accumulate, suggesting their contribution to CA alongside the aforementioned soluble sugars (Livingston et al. 2009; Yoshida 2021).

The cell wall, on the other hand, contains insoluble polysaccharides and is important as the primary line of defense against extracellular freezing. To prevent lethal freezing injury caused by extracellular ice crystals, the plant cell wall limits water movement as a physical barrier, which significantly contributes to inhibiting ice formation. Thus, the cell wall serves in a protective function against various stressors (Panter et al. 2020; Takahashi et al. 2021). Wheat and other plants of the Poaceae family contain type II cell walls made up of cellulose interlocked with hemicellulose, represented by glucuronoarabinoxylans (GAX), mixed linkage β-glucan, and trace amounts of pectin (Hatfield et al. 2017). Although cell wall thickness (Baldwin et al. 2014; Liu et al. 2022; Tanino et al. 2013) and overall cell wall content (Takahashi et al. 2019) increase during CA in various plant species such as *Allium fistulosum*, pea, and *Arabidopsis*, the detailed compositional changes in the wheat cell wall have not been clarified. On the other hand, lignin is another important secondary cell wall component that is relatively abundant in plants after cellulose. Changes in lignin content can also occur when plants grow at low temperatures (Moura et al. 2010). Lignin synthesis may contribute to cell wall modification to prevent freezing damage and cell collapse. In fact, under low positive temperatures, lignin content was found to be increased in the crown part, but unchanged in wheat leaves (Olenichenko and Zagoskina 2005). By contrast, in the case of *Arabidopsis thaliana* (Ji et al. 2015) and *Miscanthus* ecotypes (Domon et al. 2013), no obvious changes were observed in the lignin content during CA. Therefore, the relationship of lignin and freezing tolerance during CA in plants is elusive.

For the present study, we first analyzed the wheat cultivars, Yumechikara and Norin-61, which are winter cultivars, and Haruyokoi which is a spring cultivar. Yumechikara is the most late-maturing cultivar, whereas Haruyokoi is an early-maturing cultivar. Consequently, the grain yield is also higher in Yumechikara. Norin-61 and Haruyokoi have similar mean plant height, but the growth duration is longer in Norin-61, although they have similar grain yield (Supplementary Table S1) (Miyawaki et al. 2013; Tabiki et al. 2011; Tanio et al. 2005; Yanagisawa and Tabiki 2002). We observed different levels of freezing tolerance in these cultivars. To understand the mechanism for the development of freezing tolerance during CA, we analyzed the compositional and structural changes in both soluble sugars and cell wall polysaccharides during CA.

## Materials and methods

### Growth condition of wheat cultivars

**Figure 1:**
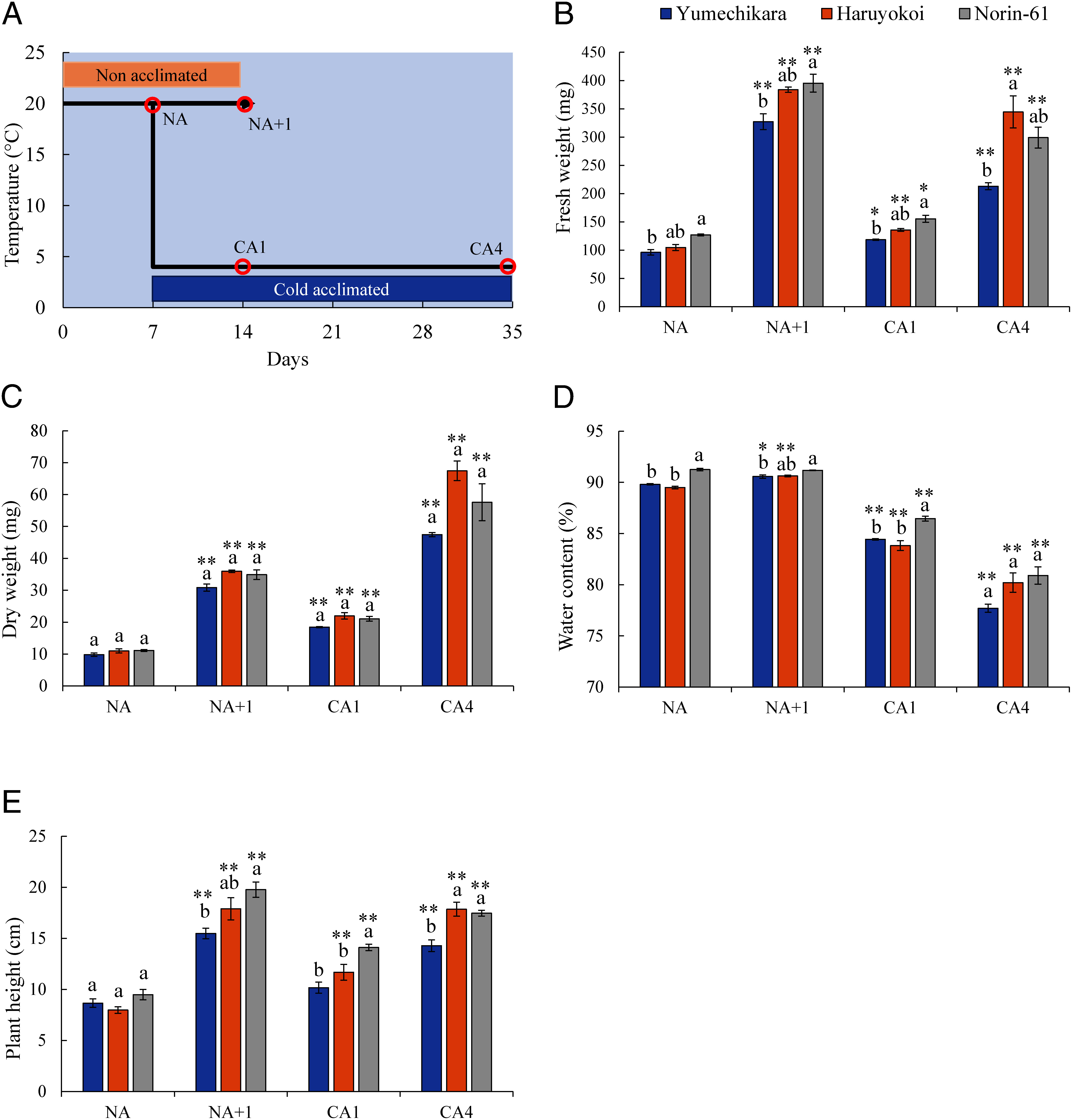
Figure 1. Experimental design and changes in the growth of three wheat cultivars. (A) Two treatments, producing NA and CA plants were used. Starting point was NA which was 7 days old seedlings grown at 20°C. NA+1 plants were grown for 1 more week at the same temperature. CA plants were treated at 4°C for 1 week (CA1) and 4 weeks (CA4). Here, NA+1 served as the control for plant growth at the CA4 stage. (B) Changes in levels of fresh weight. (C) Changes in levels of dry weight. (D) Changes in levels of water content in the three wheat cultivars. (E) Difference in plant height between the three wheat cultivars under different acclimation treatments. Seedling height was calculated using ImageJ 1.53c. Error bars indicate ±SEM (*n*=5). Significant differences computed using Tukey’s HSD test for cultivars at the same acclimation point are indicated by different letters. Statistical differences with NA and other acclimation points were determined with Dunnett’s test (* *p*<0.05, ** *p*<0.01).

Three wheat cultivars, Yumechikara, Haruyokoi, and Norin-61, were used in this study (Supplementary Table S1). Seeds of the cultivars were sown in tray pots composed of vermiculite and pearlite at a ratio of 2 : 1. For plant nutrition, the liquid fertilizer Hyponex (Hyponex Japan, Osaka, Japan), which is widely used in laboratory plant cultivation, was supplied every few days. Two sets of plants were grown under light and dark conditions (16/8 h light/dark, 120 µmol m^−2^ s^−1^) for 7 days at 20°C, designated as non-acclimated (NA) plants. Then the plants were transferred to CA condition, where they were grown at 4°C under light and dark conditions (12/12 h light/dark, 120 µmol m^−2^ s^−1^) for 7 days (CA1) and 28 days (CA4). NA plants were further cultivated for 7 days, designated as NA+1 (Figure 1A). Here the NA+1 plants were used as a developmental control for CA4. These time and temperature conditions were selected since most winter crops, including wheat, acquire freezing tolerance at about 28–56 days under low temperatures between 0°C to 10°C (Fowler et al. 1996; Laudencia-Chingcuanco et al. 2011). Leaves (the above ground part) and the crown (1 cm length from the base) (Supplementary Figure S1) were used to evaluate freezing tolerance and extract soluble sugars and cell polysaccharides. The number of replicates was three unless stated otherwise.

### Measurement of seedling height

Wheat plants used for height measurements were grown under the same conditions as described in Growth condition of wheat cultivars. Intact plants were sampled randomly at NA, NA+1, CA1 and CA4 (Figure 1A), and above-ground parts of each cultivar were photographed with a digital camera. Later, the seedling height of the cultivars was measured using ImageJ 1.53c.

### Electrolyte leakage assay

The electrolyte leakage (EL) assay was conducted following the protocol described by Takahashi et al. (2013) and Webb et al. (1994). Test tubes were filled with 100 µl of distilled water (DW), and plant samples of leaves or crown were placed inside the tubes separately as shown in Supplementary Figure S1. It was ensured that all plant parts were immersed at the bottom of the tube except for the liquid nitrogen and unfrozen control tubes. The rest of the test tubes were subjected to cooling in a programmable freezer (NCB-3300, EYELA, Tokyo, Japan). First, they were equilibrated at −2°C for 30 min, then ice particles were applied uniformly, and incubation proceeded for 60 min. Subsequently the temperature of the programmed freezer was reduced at a rate of 2°C h^−1^. At −2, −4, −6, −8, −10, −12 and −14°C, test tubes were transferred to a 4°C refrigerator for thawing overnight for up to 12 h. Next, 3 ml of water was added to each tube, and the tubes were shaken for 2 h. After measuring the electrical conductivity with a compact electrical conductivity meter (LAQUAtwin EC-33B, HORIBA, Kyoto, Japan), samples were boiled for 15 min, and the electrical conductivity value was again measured. The loss of electrolytes was calculated by the ratio of EL before and after boiling, expressed as EL percent.

### Cell wall fractionation and sugar analysis

Plants were sampled at NA, NA+1, CA1 and CA4 time points as described in Growth condition of wheat cultivars (Figure 1A). The upper surface part of the leaves and crown part (Supplementary Figure S1) were harvested and ground with a mortar and pestle. Soluble, starch, hot water, EDTA, KOH, and cellulose fractions were extracted in accordance with the method illustrated by Kutsuno et al. (2023). Total sugar composition and quantity of the above fractions were determined using the phenol sulfuric acid procedure (DuBois et al. 1956). Reducing sugars were determined from the soluble fractions by the neocuproine test (Dygert et al. 1965). The exact workflow of the fractionation process is described in Supplementary Figure S2. Fructan amount was determined using a fructan assay kit (K-FRUC, Megazyme, Dublin, Ireland) from aliquot amounts of the soluble fraction (40 µl of NA and 20 µl of CA for leaves, 20 µl of NA and 8 µl of CA for crown).

EDTA and KOH fractions were dialyzed for two days against distilled water. The samples were lyophilized and dissolved with 2N trifluoracetic acid (TFA), then hydrolyzed into monosaccharides at 120°C for 60 min. The hydrolyzed samples were then dried with a Speed Vac (VC-360, TAITEC, Saitama, Japan) to remove TFA. The resultant hydrolysate was analyzed by high-performance anion exchange chromatography-pulsed amperometric detection (HPAEC-PAD) with the DIONEX ICS-5000^+^ series system equipped with a CarboPac PA-1 column (Thermo Fisher Scientific, Waltham, MA, USA). Elution was performed using water, 0.1 M sodium hydroxide, and 0.5 M sodium acetate (Sawake et al. 2015).

### Lignin quantification

Lignin content was measured as in the protocol described by Jin et al. (2007) with some modifications. Fresh leaf and crown samples were ground and treated with 80% ethanol for 15 min. Precipitate was treated with 3 M HCl and mercaptoacetic acid at 80°C for 3 h. This was then washed and supplemented with 1 M NaOH and put on a shaker for 16 h. 12 M HCl was added to the collected supernatant and the mix was incubated at 4°C for 4 h. After incubation, 1 M NaOH was added to precipitates and the absorbance was measured at 280 nm. Lignin (dealkaline) was used as the standard for quantifying the lignin in the samples.

### Determination of enzymatic activity

For enzyme assays, wheat plants (fresh weight, 0.2 g) from NA, NA+1, CA1 and CA4 were homogenized with a mortar and pestle in homogenization buffer containing 50 mM Tris-HCl buffer (pH 7.0). The homogenate was centrifuged to remove cell debris and then used as the crude enzyme fraction. The reaction mixture contained 50 mM Tris-HCl buffer (pH 7.0), and 10 mg ml^−1^ Suc as a substrate for invertase, or 10 mg ml^−1^ starch as a substrate for amylase. Incubation for 6 and 3 h at 37°C was applied for the invertase and amylase reaction, respectively. After incubation, the reducing sugars were quantified by the neocuproine method (Dygert et al. 1965). One unit of enzyme activity liberates 1 mol of reducing sugar per minute. Activity was expressed as milliunits per gram fresh weight (mU g^−1^ FW). Protein concentration in the samples was determined as described by Bradford (1976) with bovine serum albumin (BSA) as the standard.

### RNA extraction and RT-qPCR analysis

Total RNA was extracted using Isogen (Nippon Gene, Tokyo, Japan) according to manufacturer’s instructions. RNA samples (1 µg) were converted to cDNA with oligo dT primers according to the instructions of the PrimeScript RT reagent Kit with gDNA Eraser (Takara Bio, Shiga, Japan). Quantitative PCR was performed in accordance with manufacturer’s instructions for THUNDERBIRD SYBR qPCR Mix (TOYOBO, Tokyo, Japan) using the StepOne Real-Time PCR System (Thermo Fisher Scientific). *T. aestivum tubulin* (*TaTUB*), *T. aestivum Actin* (*TaActin*) and *T. aestivum 18S rRNA* (*Ta18S rRNA*) were used as the internal standard genes. All primers used in the experiment are listed in Supplementary Table S2.

### Data analysis

Statistically significant differences of the recorded data were determined using Student’s *t*-test for two-group comparisons and Dunnett’s or the Tukey’s HSD test for multiple comparisons at the *p*<0.05 and *p*<0.01 level. These analyses were conducted using Microsoft Excel 365 (for Windows version 2008) or R (version 4.2.1) with RStudio (version 2023.12.0+369).

## Results

### Growth and electrolyte leakage depending on acclimation stages

**Figure 2:**
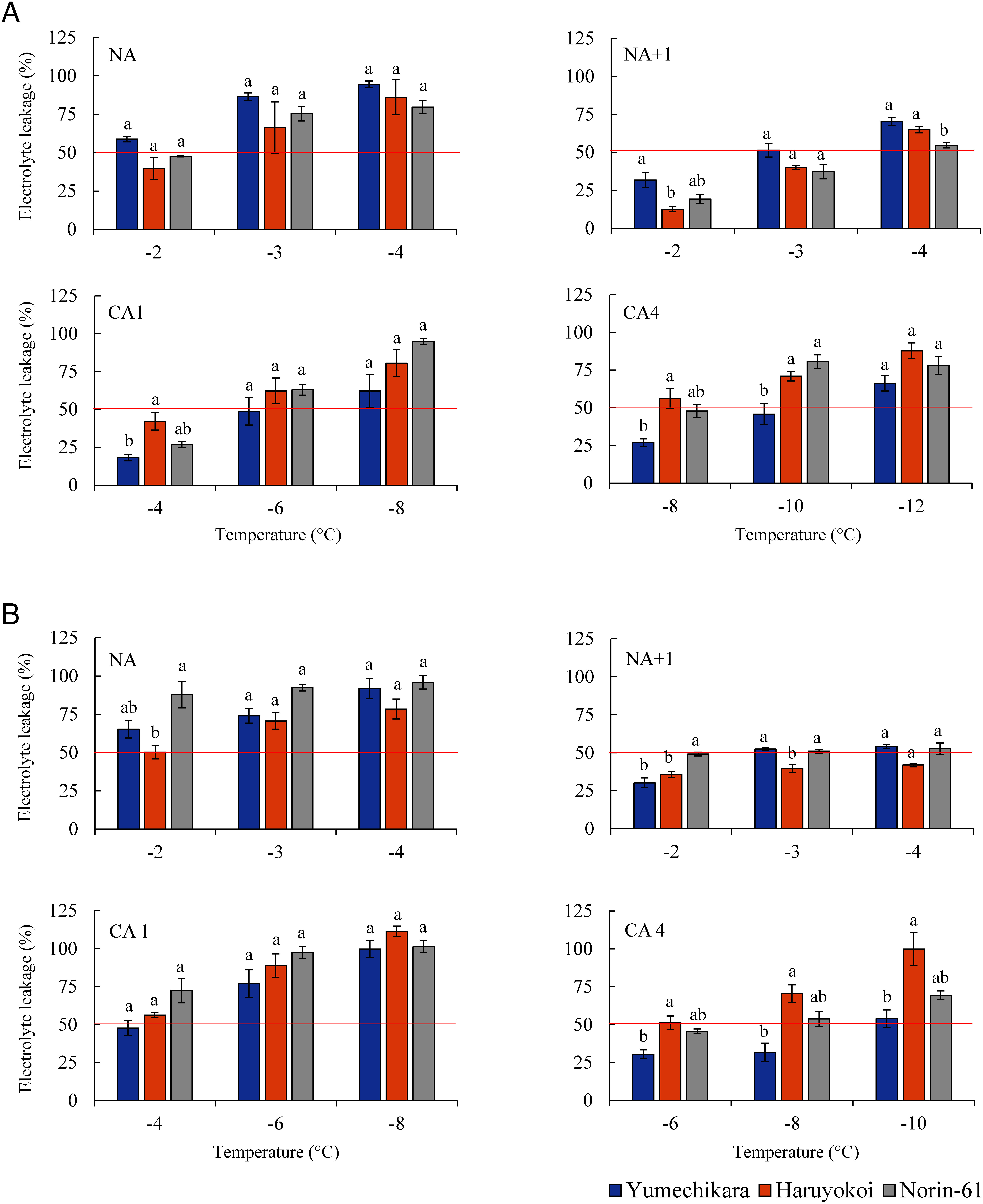
Figure 2. Electrolyte leakage (EL) in the leaves and crown part for different acclimation levels. (A) Individual leaves were cut for the electrolyte leakage assay to determine the freezing tolerance of the three wheat cultivars. (B) The crown part (excluding roots) of the wheat cultivars about 1 cm in length was excised and placed in test tubes for the EL assay to assess freezing tolerance in the crown part. Bars represent the EL% at a given temperature. Red horizontal lines indicate the 50% EL threshold where 50% of the electrolytes have leaked. Error bars indicate ±SEM (*n*=4 for leaf parts, *n*=3 for crown parts). Significant differences among cultivars at each temperature point are indicated by different letters (Tukey’s HSD test).

Plants acquire freezing tolerance after CA, while growth can be affected. Therefore, we first compared the growth of three wheat cultivars and observed poor growth of Yumechikara at CA4, whereas under NA, all cultivars exhibited similar growth. Consequently, Yumechikara showed reduced fresh weight, dry weight and water content (%) at CA4 compared to Haruyokoi (Figure 1B, C, D). The plant height of Haruyokoi at CA4 was similar to that at NA+1, but the growth of Yumechikara was slightly suppressed at CA4 compared with NA+1 (Figure 1E). To evaluate tissue damage during freezing, we performed EL assays on leaves and crown parts. The crown is important because it contains meristem tissue that may or may not survive freezing injury and thus plant survival depends on whether the crown survives. Although Yumechikara and Haruyokoi under NA and NA+1 conditions exhibited similar tissue damage in leaves and crown (Supplementary Figures S3, S4), Yumechikara leaves after CA4 showed high tolerance to freezing, as more than 54% leaf cells in this cultivar were undamaged after freezing at −10°C, while in Haruyokoi, 71% cells were damaged at the same freezing temperature. (Figure 2A). The crown part in Yumechikara also exhibited higher freezing tolerance after CA4, but under NA and NA+1 conditions, the freezing tolerance of Yumechikara was not as strong (Figure 2B). The results suggest that the visible injuries after freezing (Supplementary Figures S3, S4) were consistent with the EL results (Figure 2A, B).

### Changes in soluble sugars during CA

**Figure 3:**
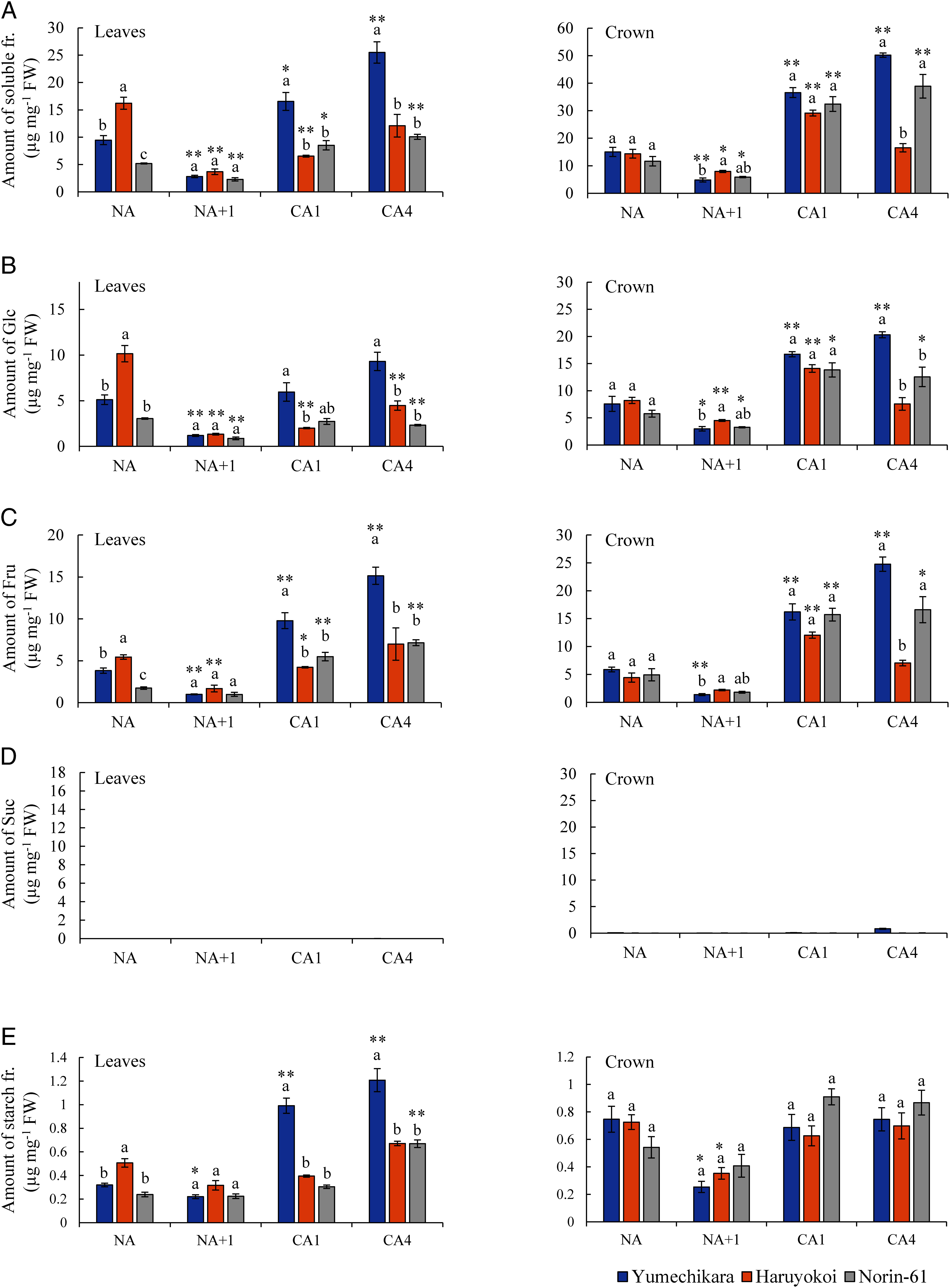
Figure 3. Changes in the amount and the composition of sugar content at different acclimation stages in leaves and crown part. (A) Soluble fraction, (B) Glc, (C) Fru, (D) Suc, and (E) starch fraction. Each row shows a specific comparison (between leaves and crown part). The Y-axis indicates the relative amount of sugar in microgram per milligram of fresh weight and the X-axis represents the four acclimation stages. Error bars indicate ±SEM (*n*=3). Significant differences, according to Tukey’s HSD test, among cultivars within the same acclimation point are indicated by different letters. Statistical differences with NA and other acclimation points were determined with Dunnett’s test (* *p*<0.05, ** *p*<0.01).

To observe the influence of CA on soluble sugars, starch, and cell wall components, we fractionated the sugars from leaves and crown parts. Soluble sugars were collected as the soluble fraction (Supplementary Figure S2). In Yumechikara leaves and crown, soluble sugars increased significantly during CA1 and CA4, with amounts significantly differing from the other two cultivars. We observed that without CA Yumechikara does not increase soluble sugars, which in fact decrease from NA to NA+1 (Figure 3A). Next, to identify the sugar composition, we analyzed the soluble sugars using HPAEC-PAD and found that they mostly consisted of Glc and Fru (Supplementary Figure S5), with the amount of Fru being higher in both parts compared to Glc. Glc and Fru accumulation in leaves was significantly higher under CA in Yumechikara compared to Haruyokoi, whereas under NA, a higher amount of Glc and Fru was observed in Haruyokoi leaves (Figure 3B, C). Glc and Fru content in the crown of Yumechikara was almost double that in the leaves for CA4, but no cultivar-specific difference was observed at CA1, as all cultivars tended to increase Glc and Fru levels (Figure 3B, C). Remarkably, in the leaves of Norin-61, Glc content significantly reduced during CA4, but in the case of the crown, Glc level increased significantly at CA4 (Figure 3B). The amount of total reducing sugars, including Glc and Fru, was elevated at CA1 and CA4 of Yumechikara (Supplementary Figure S6), consistent with the levels of Glc and Fru (Figure 3B, C). Interestingly, no Suc accumulation during CA was observed in all cultivars (Figure 3D).

After the soluble fraction, a starch fraction was also collected. Yumechikara leaves had significantly higher starch content after CA1 and CA4 than the other two cultivars, although before CA, the content was similar to that of the other cultivars (Figure 3E).

**Figure 4:**
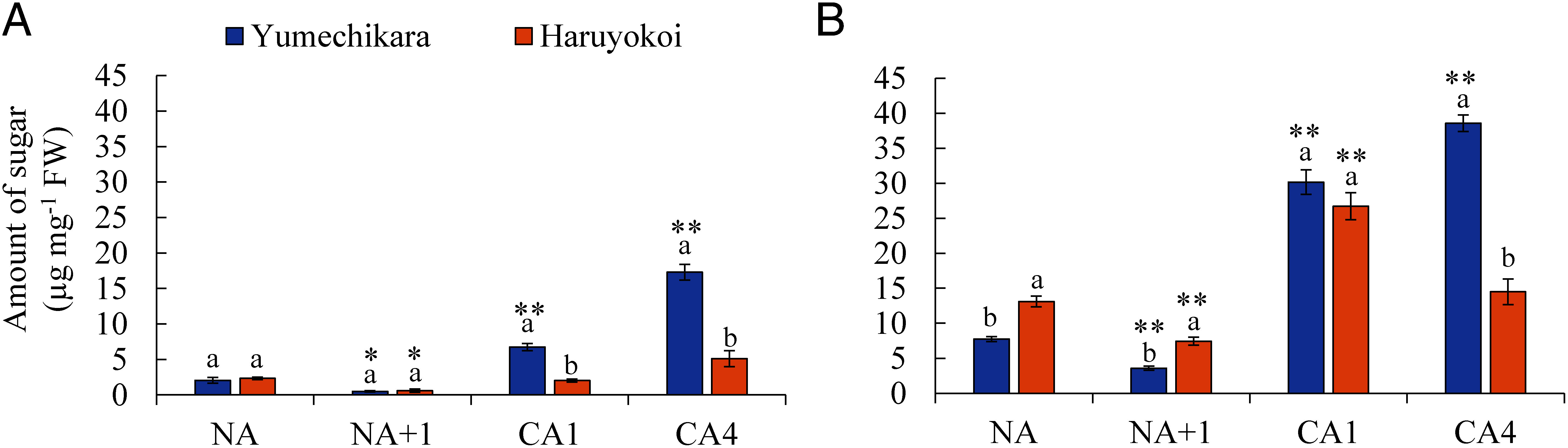
Figure 4. Fructan content in winter (Yumechikara) and spring (Haruyokoi) wheat cultivars at different acclimation points. (A) Changes in fructan amount in leaves. (B) Changes in fructan amount in the crown part. Individual rows show the part specific comparison (between leaves and crown part). The Y-axis represents fructan quantity in microgram per milligram of fresh weight and the X-axis represents the four acclimation stages. Error bars indicate ±SEM (*n*=3). Different letters indicate significant differences among cultivars at identical acclimation points according to Tukey’s HSD test. Statistical differences with NA and other acclimation points were determined with Dunnett’s test (* *p*<0.05, ** *p*<0.01).

In this study, fructan content was determined in soluble fractions extracted from leaves and crown of Yumechikara and Haruyokoi (Figure 4). The results showed that the amount of fructan in leaves increased significantly in Yumechikara during the CA process, while it was stable in Haruyokoi (Figure 4A). In the crown, Yumechikara had significantly higher amounts of fructan than Haruyokoi at CA4 (Figure 4B). This result is in perfect agreement with the results of measuring the amount of Fru in the hydrolysate of the soluble fraction (Figure 3C), indicating that a substantial amount of the Fru in the soluble fraction is derived from fructan.

### Changes in cell wall polysaccharides and lignin during CA

**Figure 5:**
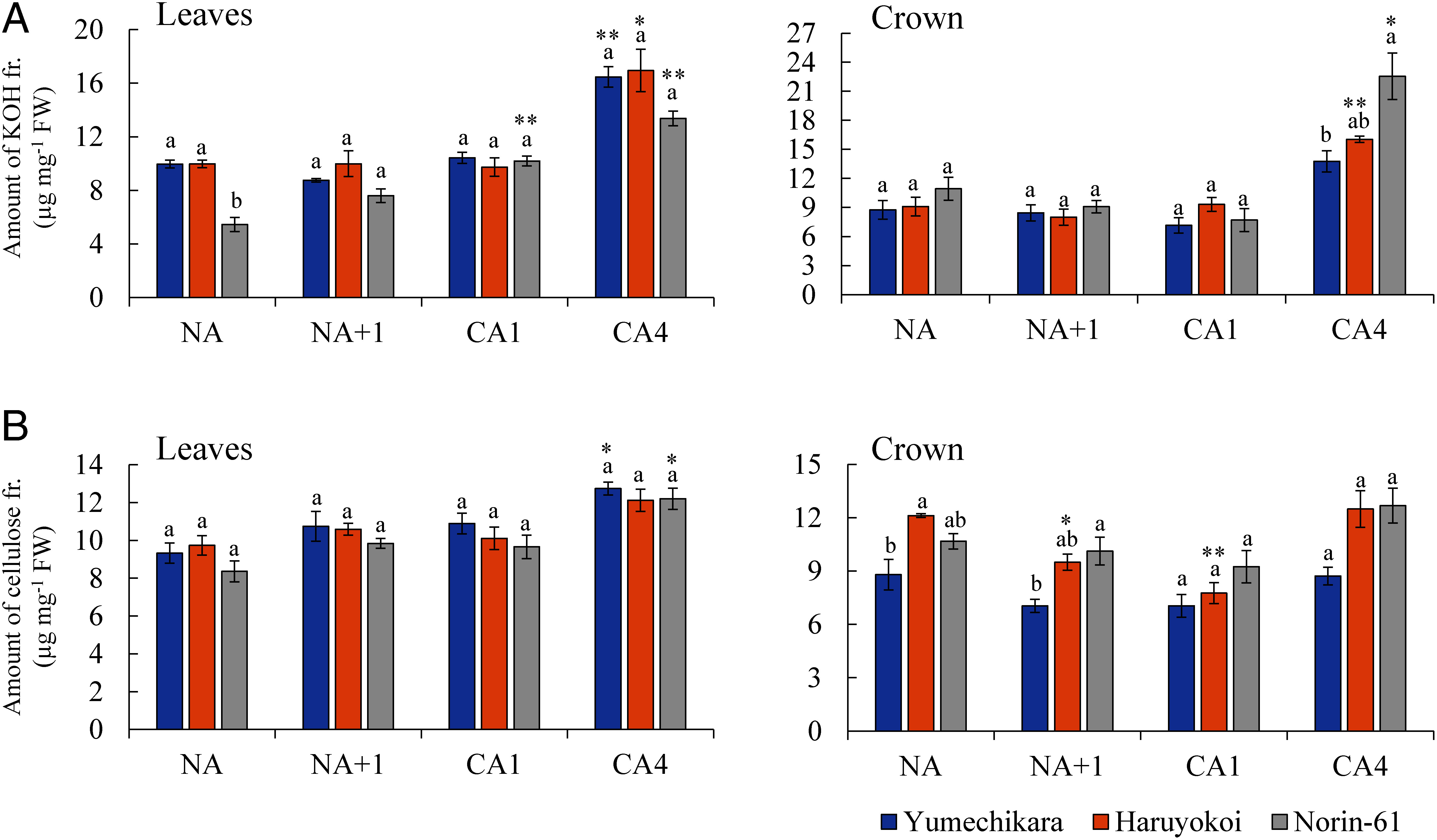
Figure 5. Changes in total sugar content of cell wall fractions at different acclimation stages in leaves and crown part. (A) KOH fraction and (B) cellulose fraction. Y-axis: microgram sugar per milligram of fresh weight; X-axis: the four acclimation stages. Error bars indicate ±SEM (*n*=3). Significant differences among cultivars from the same acclimation regime are indicated by different letters (Tukey’s HSD test). Statistical differences with NA and other acclimation points were determined with Dunnett’s test (* *p*<0.05, ** *p*<0.01).

Following the extraction of soluble and starch fractions, cell wall polysaccharides were also fractionated into a hot water fraction, an EDTA fraction rich in pectin, then a KOH fraction rich in hemicellulose and the residual cellulose fraction (Supplementary Figure S2). From the results, trace amounts of pectin enriched in the hot water and EDTA fractions were detected (Supplementary Figure S7). With the influence of CA, all cultivars tended to increase the hemicellulose content in leaves and crown at CA4, but no significant differences among the cultivars were observed (Figure 5A). Furthermore, no obvious difference in cellulose content among cultivars was observed in leaves or crown irrespective of CA (Figure 5B).

**Figure 6:**
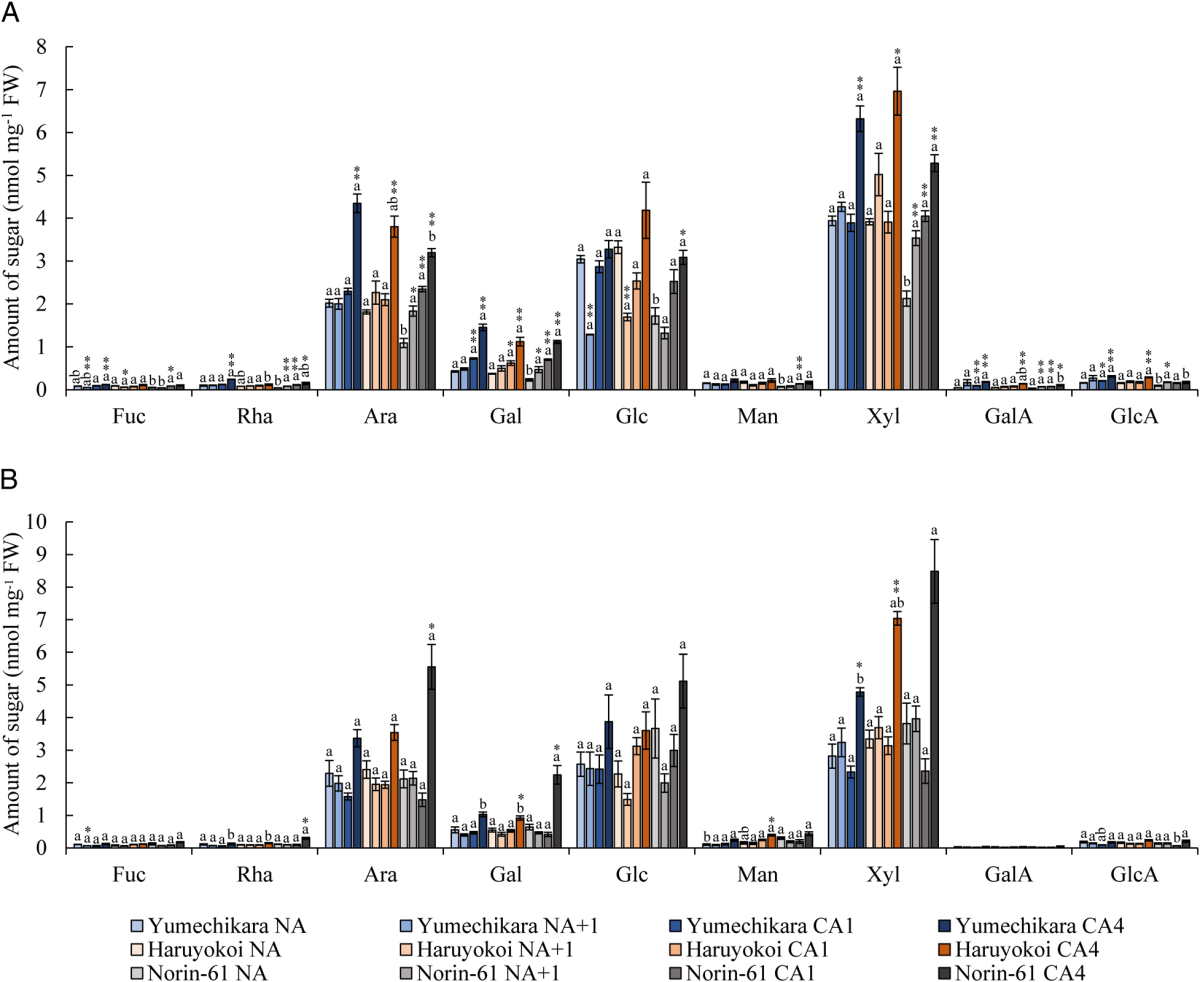
Figure 6. Constituent sugar composition of the KOH fraction derived from NA and CA samples of (A) leaves and (B) crown. The graphs present the amount of each monosaccharide on a per milligram fresh weight basis. Monosaccharide compositional analysis was performed using HPAEC-PAD. Error bars represent ±SEM (*n*=3). Significant differences among cultivars within acclimation points are indicated by different letters (Tukey’s HSD test). Statistical differences with NA and other acclimation points were determined with Dunnett’s test (* *p*<0.05, ** *p*<0.01).

Next, to identify the changes in hemicellulose during CA, we determined the sugar composition of the KOH fraction. In wheat the major hemicellulosic polysaccharide is arabinoxylan. Abundant Ara and Xyl were detected together with Glc, which probably constitutes mixed linkage β-glucan (Figure 6). In leaves, no clear difference in monosaccharides derived from hemicellulose among the cultivars was observed at CA4, but a significant increase of Ara and Xyl was seen at CA4 in all cultivars (Figure 6A). In the crown part, Xyl content was significantly increased at CA4 in all cultivars, where crown tissue in Yumechikara has a smaller amount of Xyl than Haruyokoi (Figure 6B). However, there was no correlation between the freezing tolerance of each cultivar and the constituent sugars of hemicellulose in either leaves or crown.

As lignin is another important secondary cell wall component, we also examined the extent of changes in lignin content during CA and found that the amount of lignin was much less (Supplementary Figure S8) than the amount of hemicellulose and cellulose (Figure 5). Moreover, changes in the lignin content during CA and differences among cultivars were not at all obvious in either leaves or crown (Supplementary Figure S8).

### Changes in enzymatic activity and gene expression

**Figure 7:**
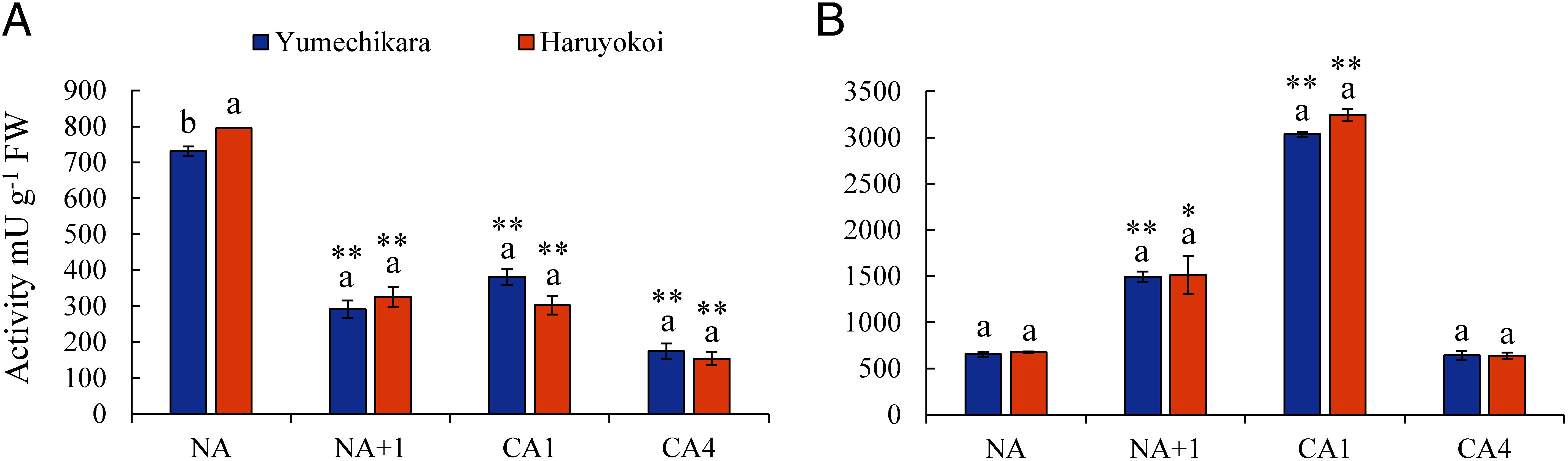
Figure 7. Enzymatic activity in wheat cultivars at different acclimation times. (A) Invertase, (B) amylase activity in milliunit per gram of fresh weight. Freshly harvested plants were used to measure the enzyme activity and protein content in two wheat cultivars. Error bars indicate ±SEM (*n*=3). Significant differences among cultivars within the acclimation points are indicated by different letters (Tukey’s HSD test). Statistical differences between NA and other acclimation points were determined with Dunnett’s test (* *p*<0.05, ** *p*<0.01).

This study confirmed a significant CA-induced increase in Glc and Fru in the soluble fraction, especially in the freeze-tolerant cultivar Yumechikara (Figure 3B, C). Therefore, we quantified the activities of invertase and amylase based on fresh weight in Yumechikara and Haruyokoi during the CA process (Figure 7). Invertase activity at NA+1 and CA4 was significantly lower than at NA in both cultivars, indicating that Suc may have been consumed during normal growth. In addition, invertase activity was not significantly different in either cultivar under any acclimation condition (Figure 7A). Amylase activity at CA4 was similar to that at NA in both cultivars, implying that Glc derived from starch degradation peaked at CA1, and at CA4, the NA Glc level was restored (Figure 7B). Additionally, invertase and amylase activity based on protein content of Yumechikara and Haruyokoi (Supplementary Figure S9) were rather similar to activity based on fresh weight.

**Figure 8:**
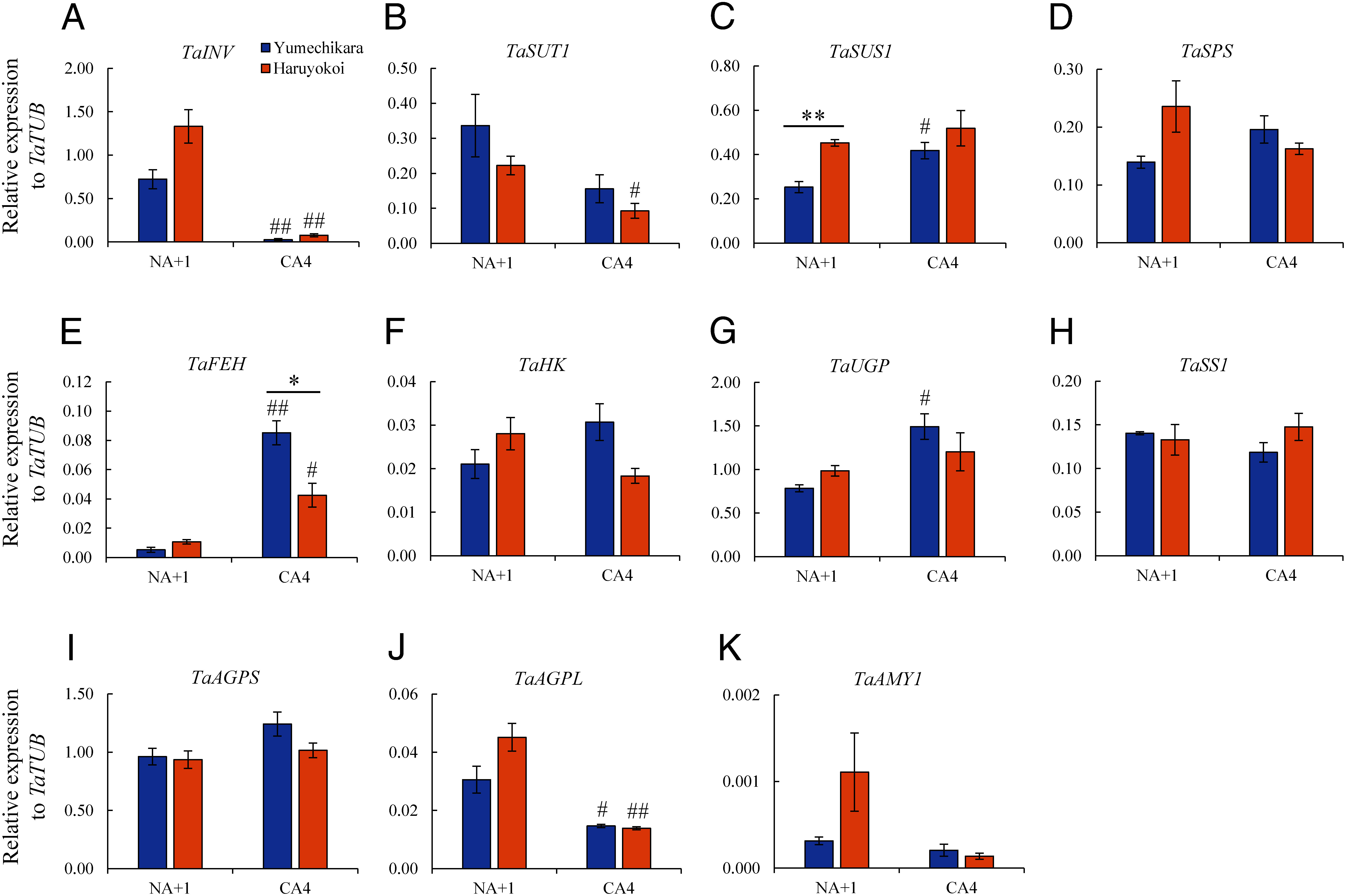
Figure 8. Differential expression level of starch and Suc metabolism enzymes in Yumechikara and Haruyokoi under NA and CA. Expression pattern of (A) *TaINV*, (B) *TaSUT1*, (C) *TaSUS1*, (D) *TaSPS*, (E) *TaFEH*, (F) *TaHK*, (G) *TaUGP*, (H) *TaSS1*, (I) *TaAGPS*, (J) *TaAGPL*, (K) *TaAMY1*. Values represent the expression levels relative to that of *TaTUB.* Error bars indicate ±SEM (*n*=3). Significant differences (*t*-test) are indicated by asterisks among the bars and hash (#) symbols indicate comparison with NA+1 (*/^#^ *p*<0.05, **/^##^ *p*<0.01).

To confirm the enzymatic activities, we examined the expression of related enzyme genes involved in Glc and Fru metabolism (Supplementary Figure S10). For this investigation, we selected *T. aestivum hexokinase* (*TaHK*), *T. aestivum Suc transporter 1* (*TaSUT1*), *TaSUS1*, *TaSPS*, *T. aestivum fructan 6-exohydrolase* (*TaFEH*), *T. aestivum UDP-Glc pyrophosphorylase* (*TaUGP*), *TaSS1*, *TaAGPS*, *TaAGPL*, in addition to *TaINV* and *TaAMY1*, and performed gene expression analysis by RT-qPCR in Yumechikara and Haruyokoi (Figure 8). Expression levels of *TaINV* markedly reduced during CA, but no changes in *TaAMY1* were observed during CA (Figure 8A, K and Supplementary Figures S11A, K, S12A, K). This result was consistent with observed enzyme activity (Figure 7). Starch accumulated during CA in Yumechikara, and gene expression of *TaHK* and *TaUGP*, which are indirectly involved in starch synthesis, tended to increase during CA in Yumechikara (Figure 8F, G, Supplementary Figures S11F, G, S12F, G). However, the expression of *TaSS1*, which is directly involved in starch synthesis, did not as clearly increase at CA4 among the cultivars relative to *TaTUB* expression (Figure 8H), suggesting that homologs other than genes analyzed in this study may contribute to starch synthesis in CA. But with *TaActin* and *Ta18S rRNA*, the expression of *TaSS1* tended to increase in Yumechikara during CA (Supplementary Figures S11H, S12H) which was consistent with the results on starch content (Figure 3E). Also noteworthy in this study was the increased expression of *TaFEH*, which is involved in the degradation of fructan, during the CA process. Moreover, the upregulation of *TaFEH* during CA was more pronounced in Yumechikara than in Haruyokoi (Figure 8E and Supplementary Figures S11E, S12E).

## Discussion

### Effect of CA on growth and freezing tolerance

The winter cultivar Yumechikara exhibited poorer growth than the spring cultivar Haruyokoi and another winter cultivar, Norin-61. Here a trade-off relationship between growth and freezing tolerance was observed among the cultivars. Specifically, Yumechikara demonstrated higher freezing tolerance, but could not achieve better vegetative growth than Haruyokoi and Norin-61. The difference in plant growth may be attributable to the cultivar traits and the acclimation regime used in the experiment (Jaškūnė et al. 2022). It has been previously well-documented that the accumulation of soluble sugars during the CA process can enhance freezing tolerance (Chen and Murata 2008; Gusta et al. 2004; Hare et al. 1998; Iba 2002; Wang et al. 2003). This suggests that high amounts of soluble sugars accumulated in freezing-tolerant Yumechikara probably due to the reduced growth, whereas freezing-sensitive Haruyokoi attained vigorous growth while consuming more sugars.

### Differences in soluble sugars, enzyme activity and gene expression during CA

Accumulation of soluble sugars during CA is a fundamental phenomenon. We observed a higher accumulation of soluble sugars in Yumechikara compared to Haruyokoi after CA1 and CA4. CA1 is insufficient for full sugar accumulation, which is completed only at CA4. It stands to reason that the high accumulation of soluble sugars in Yumechikara compared to Haruyokoi was the reason for reduced freezing injury in the former, as these sugars are known to act as cryoprotectants (Garg et al. 2002) and both leaves and crown of Yumechikara contained higher amounts of Glc and Fru than Haruyokoi during CA4. Glc can be generated from both starch and Suc, but here increased Glc presumably mainly originated from starch, as the level of Suc we detected was very low. The severely limited amount of Suc accumulation contrasts with prior studies that have shown that Suc increased after prolonged CA conditions in several wheat cultivars (Larsson et al. 1992) and might be due to the growth conditions we used, for example, light intensity (Gusta and Wisniewski 2013) and/or type and amount of fertilizer. Indeed, plants growing in the field under natural conditions are exposed to 4–12 times stronger light than plants growing in laboratory condition (Gusta and Wisniewski 2013), and low-light environments dynamically alter the metabolome including Suc metabolism in wheat (Yang et al. 2021). Furthermore, it has been shown in several plants that nutritional status affects carbon assimilation during CA process (Martindale and Leegood 1997) and ultimately influences freezing tolerance (Hetherington et al. 1989).

Although our data confirmed the higher accumulation of Glc and Fru in Yumechikara during CA, *TaINV* and *TaAMY1* gene expression was reduced during CA and no difference in enzyme activity between the cultivars was observed. Plants increase Glc concentrations as it is generated from starch and Suc by amylase and invertase, respectively. Fru is also produced by the degradation of Suc by invertase. The drastic reduction of invertase activity in CA4 compared to NA+1 may be attributable to reduced Suc accumulation at low temperatures, which may inhibit invertase activity, as previously reported in wheat (Shahryar and Maali-Amiri 2016), *Arabidopsis thaliana* (Klotke et al. 2004), maize (Boyer and McLaughlin 2007), and *Camellia sinensis* (Yao et al. 2020). The exact reason for the reduced α-amylase activity and *TaAMY1* expression in CA4 is not clear. Previous studies have shown increased α-amylase activity in potato leaves during cold stress (Sitnicka and Orzechowski 2014). Therefore, changes in amylase activity at low temperatures may depend on the plant species and/or growth environment. Overall, Yumechikara did not show activity of invertase or amylase distinct from Haruyokoi, suggesting that there may be unknown mechanisms involved in increasing Glc and Fru to different levels in these cultivars.

In Poaceae plants, fructan accumulation is known to occur in CA, suggesting that it is involved in freezing tolerance through membrane stabilization (Livingston et al. 2009). Although Fru can be generated from Suc, Fru may also have originated from fructan (Supplementary Figure S10). To investigate this, gene expression of *TaFEH* was checked and found to significantly increase in Yumechikara by CA4 but not in Haruyokoi. The significantly higher *TaFEH* expression level in Yumechikara during CA4 suggests that this winter cultivar can generate a larger amount of Fru from fructan degradation by the action of FEH enzymes (Van den Ende et al. 2004) during CA than the spring cultivar Haruyokoi. This result aligns with the findings of Khoshro et al. (2014) in the ‘Zagros’ wheat cultivar, where the *TaFEH* expression increased during drought. On the other hand, our study also revealed a remarkable increase in fructan itself, especially in freezing tolerant Yumechikara (Figure 4). This is consistent with previous studies showing that expression levels of fructan synthesis genes correlate well with freezing tolerance in various lines of wheat (Yokota et al. 2015; Yoshida et al. 1998). The degree of polymerization (DP) of fructan is diverse from oligosaccharide to DP 100 (Yoshida 2021). Thus, with regard to fructan, one must consider that it is not simply the total amount, but also its structure (e.g., DP) that may be related to freezing tolerance (Livingston et al. 2009). Fructan is highly hydrophilic and has been shown to have membrane-protective activity in vitro (Livingston et al. 2009; Valluru and Van den Ende 2008). In addition, compared to monosaccharides such as Glc and Fru, fructan is less likely to contribute to the osmotic pressure increase that resists freeze-induced dehydration, but may also be used as energy for regrowth after overwintering (Yoshida 2021).

The source tissue, like leaves, is a part of the plant that produces nutrients, particularly sugars, through photosynthesis, whereas the sink tissue, like the crown, is a part of the plant that actively consumes or stores the sugars. In Yumechikara, significant increases during CA were observed in monosaccharides such as Glc and Fru, and in storage polysaccharides such as starch and fructan in leaves and/or crown. From our results it may be inferred that starch can be stored in the source (leaf) tissue and degraded to Glc, which then may be transported to the sink (crown) tissue and consumed during or after a spell of cold temperatures in Yumechikara. Similar events can occur in the case of fructan in Yumechikara, whereby a large amount of fructan can be accumulated in both source and sink tissues and consumed in sink tissue as Fru during or after the cold environment period. The differences in CA changes in sugar composition between freezing-tolerant and susceptible cultivars found in this study indicate that the sink-source balance and the growth-freezing tolerance tradeoff are mutually influential.

### Influence of CA on cell wall polysaccharides and lignin content

Cell wall polysaccharides contribute to increased freezing tolerance, as previously reported in *Arabidopsis* and other plants regarding changes in pectin (Chen et al. 2018; Panter et al. 2019; Takahashi et al. 2024) and hemicellulose (Takahashi et al. 2021) during CA. In the case of wheat, the main hemicellulosic polysaccharide is arabinoxylan (Izydorczyk and Biliaderis 1995), which has a backbone of β-D-xylose (Xyl) residues and also has side chains containing L-arabinose (Ara) (Carpita and McCann 2000; Scheller and Ulvskov 2010). Arabino (glucurono) xylan constitutes about 20–40% of primary cell walls in the Poaceae family (Scheller and Ulvskov 2010). We thus extracted the arabinoxylan-containing KOH fraction, as Ara and Xyl were enriched in this fraction, but did not find any significant differences among the wheat cultivars during CA. However, the amount of Ara and Xyl significantly increased during CA4, which may indicate a role for arabinoxylan in increasing basal freezing tolerance. On the other hand, another major hemicellulose, mixed linkage β-glucan, remained mostly unaffected by CA, as previously observed in *Miscanthus* clones (Domon et al. 2013). Furthermore, wheat or grass family plants contain a smaller amount of pectin (Konishi et al. 2011), comprising around 2–10% in total according to Albersheim et al. (2011), which is consistent with our results. Hence, we suggest that the contribution of pectin to the overall freezing tolerance of the wheat cultivars might be limited. However, in the future, tissue specific accumulation patterns and the cross-linking status of arabinoxylan (Willick et al. 2018) should also be analyzed to make a comprehensive judgment. Lignin accumulation may be different based on plant species and tissue type under low temperatures (Moura et al. 2010). Previously Ji et al. (2015) have reported that reduced lignin content increased freezing tolerance in *Arabidopsis*. But in case of the wheat cultivars we used, this relationship was not apparent.

In summary, sugar utilization during CA greatly varied among the wheat cultivars, influencing growth and freezing tolerance. On the other hand, changes in insoluble cell wall polysaccharides during CA may not be strongly associated with cultivar specific freezing tolerance during CA. We found that the accumulation of soluble sugars was different between the source (leaves) and the sink (crown) tissue, indicating that sugar synthesis and utilization are not the same in these two parts of wheat plants. Although invertase and amylase activity and gene expression were very low in CA4, and no difference was seen between Yumechikara and Haruyokoi, the concentration of soluble sugars (Glc and Fru) was high at CA4 in Yumechikara, which may be due to some unknown pathways, factors, or mechanisms. Changes in storage polysaccharides such as starch and fructan closely correlated with the accumulation pattern of simple sugars such as Glc and Fru. Both are thought to contribute deeply to the freezing tolerance acquired after CA in tolerant cultivars like Yumechikara through complex regulation involving sink-source dynamics and growth/freezing tolerance tradeoffs.

## Abbreviations

Ara: arabinose
CA: cold acclimation
EL: electrolyte leakage
Fru: fructose
Fuc: fucose
FW: fresh weight
Gal: galactose
GalA: galacturonic acid
Glc: glucose
GlcA: glucuronic acid
HPAEC-PAD: high-performance anion-exchange chromatography with pulsed amperometric detection
Man: mannose
NA: non-acclimated
Rha: rhamnose
Suc: sucrose
*TaAGPL*: *Triticum aestivum ADP-Glc pyrophosphorylase large subunit*
*TaAGPS*: *T. aestivum ADP-Glc pyrophosphorylase small subunit*
*TaAMY1*: *T. aestivum amylase*
*TaFEH*: *T. aestivum fructan exohydrolase*
*TaHK*: *T. aestivum hexokinase*
*TaINV*: *T. aestivum invertase*
*TaSPS*: *T. aestivum Suc phosphate synthase*
*TaSS1*: *T. aestivum starch synthase*
*TaSUS1*: *T. aestivum Suc synthase*
*TaSUT1*: *T. aestivum Suc transporter*
*TaTUB*: *T. aestivum tubulin*
*TaUGP*: *T. aestivum UDP-Glc pyrophosphorylase*
Xyl: xylose

## Description of Supplementary Files

Supplementary Figure S1. Plant parts used for experimentation.

Supplementary Figure S2. Flow chart of cell wall fractionation and sugar quantification by the phenol sulfuric acid method.

Supplementary Figure S3. Grow out tests of the three wheat cultivars.

Supplementary Figure S4. Morphological changes in the crown parts after freeze-thawing in plants undergoing different acclimation stages (NA, NA+1, CA1, CA4) of Yumechikara, Haruyokoi and Norin-61 wheat crowns.

Supplementary Figure S5. Ratio of constituent sugars in soluble fraction derived from NA and CA samples.

Supplementary Figure S6. Reducing sugar in wheat cultivars after different acclimation times.

Supplementary Figure S7. Changes in total sugar content of cell wall polysaccharides at different acclimation stages in leaves and crown parts.

Supplementary Figure S8. Changes in lignin content of the three wheat cultivars during cold acclimation.

Supplementary Figure S9. Activity based on protein content in wheat cultivars for different acclimation times.

Supplementary Figure S10. Possible pathways of Glc and fru metabolism in Yumechikara and Haruyokoi.

Supplementary Figure S11. Differential expression level of starch and Suc metabolism enzymes in Yumechikara and Haruyokoi under NA and CA expression levels relative to that of *TaActin*.

Supplementary Figure S12. Differential expression level of starch and Suc metabolism enzymes in Yumechikara and Haruyokoi under NA and CA expression levels relative to that of *Ta18S rRNA*.

Supplementary Table S1. Features of the studied wheat cultivars.

Supplementary Table S2. List of primers used for RT-qPCR.
