# Supplementary material for: Soluble sugars make a greater contribution than cell wall components to the variability of freezing tolerance in wheat cultivars
Source: Plant Biotechnol (Tokyo). 2024 Dec 25;41(4):401–15. doi: 10.5511/plantbiotechnology.24.0801a (PMC11897726; doi:10.5511/plantbiotechnology.24.0801a)
Supplement: Supplementary Data [file plantbiotechnology-41-4-24.0801a-s001.pdf]

Supplementary Files

Soluble sugars make a greater contribution than cell wall components to the variability of freezing tolerance in wheat cultivars

Sushan Chowhan, Takuma Kikuchi, Momoka Ohashi, Tatsuya Kutsuno, Hiroto Handa, Toshihisa Kotake, Daisuke Takahashi*

*Corresponding author: dtakahashi@mail.saitama-u.ac.jp

This pdf file includes
Supplementary Figure S1 to S12
Supplementary Table S1 to S2
Supplementary references

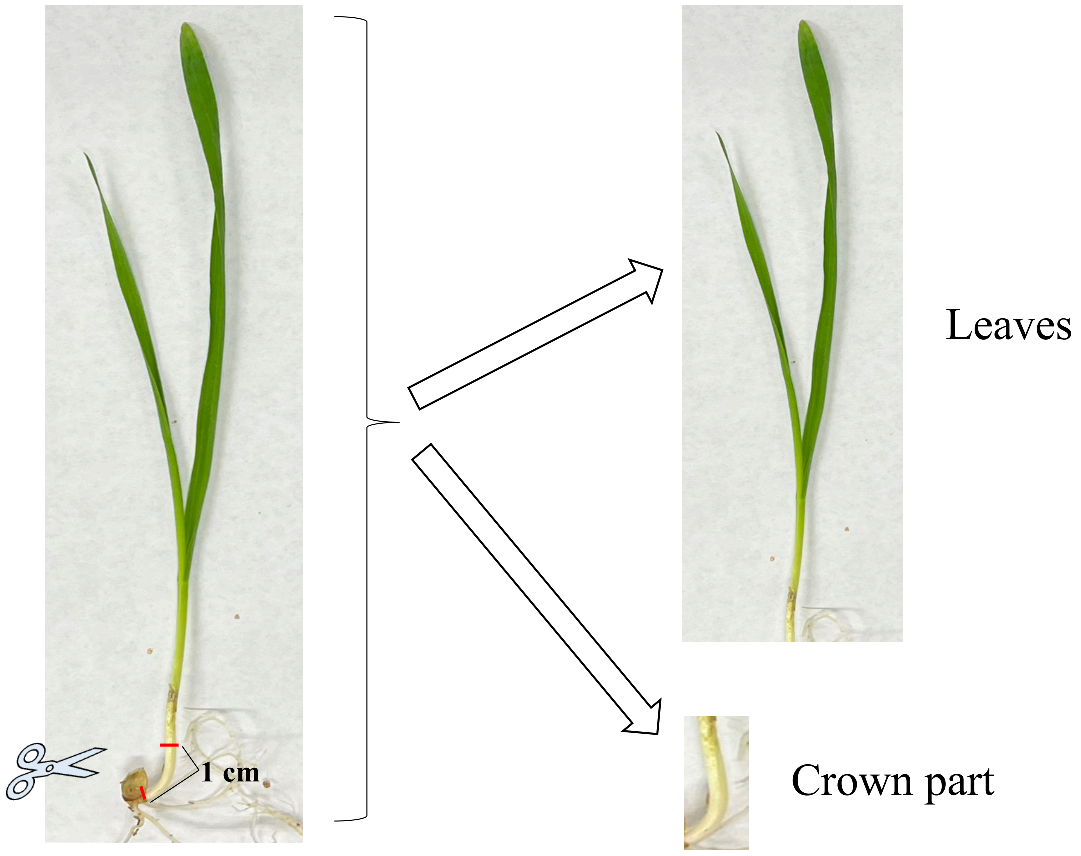

Supplementary Figure S1. Plant parts used for experimentation. Two different parts of the plants were sampled. For leaves, only leaf (excluding 1 cm from the base) parts were cut and for the crown part a segment of 1 cm in length from the base (excluding roots) was excised.

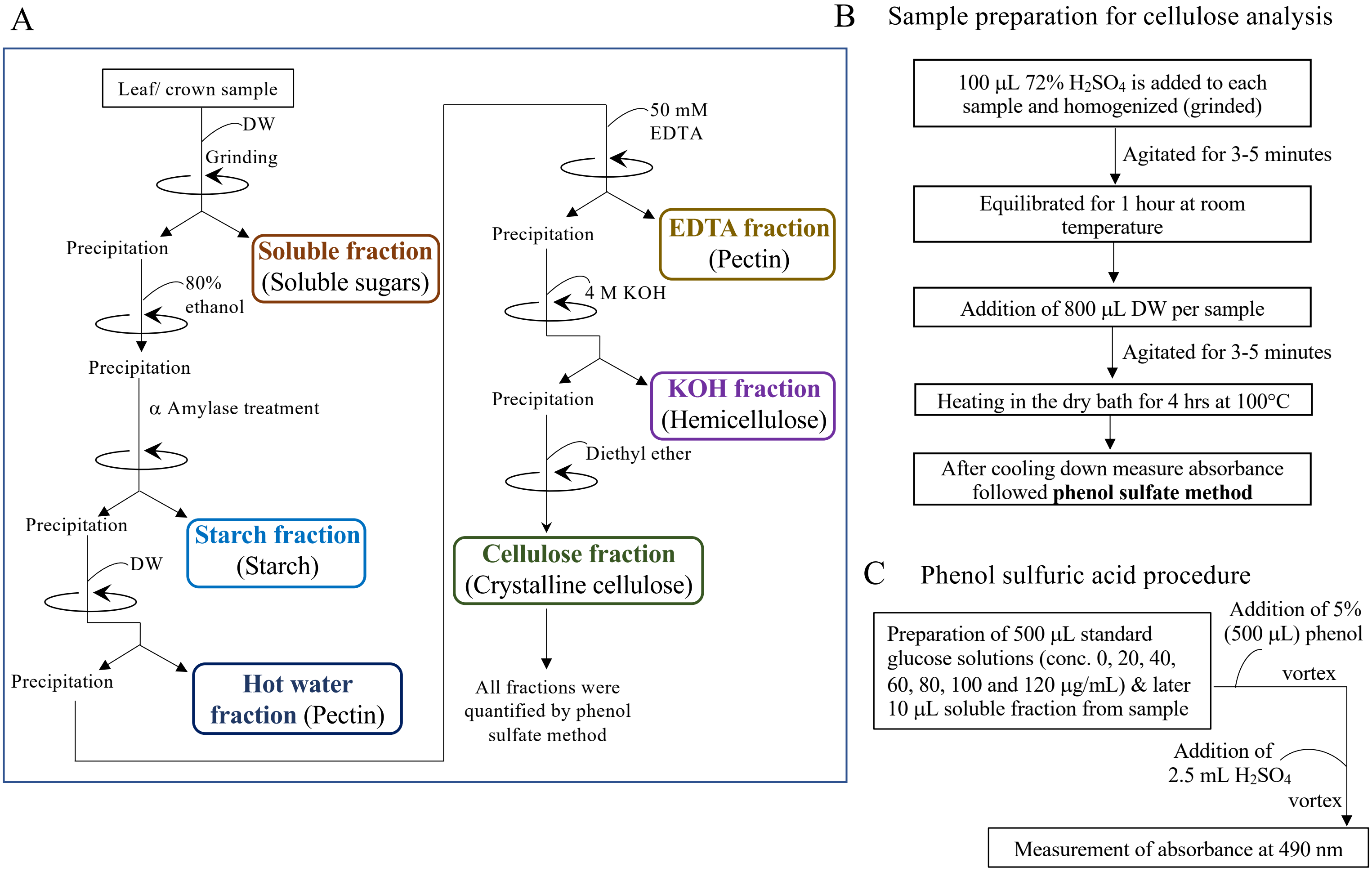

Supplementary Figure S2. Flow chart of cell wall fractionation and sugar quantification by the phenol sulfuric acid method. (A) Samples of leaves or crown were ground to extract soluble sugars, starch, pectin, and hemicellulose using α-amylase solution, hot water, EDTA solution and KOH solution. Residual parts are mainly crystalline cellulose. (B) Cellulose was hydrolyzed with sulfuric acid before inception of the phenol sulfuric acid procedure. (C) Different concentrations of glucose solutions and fractionated samples were prepared prior to phenol and sulfuric acid treatment and subjected to absorbance reading.

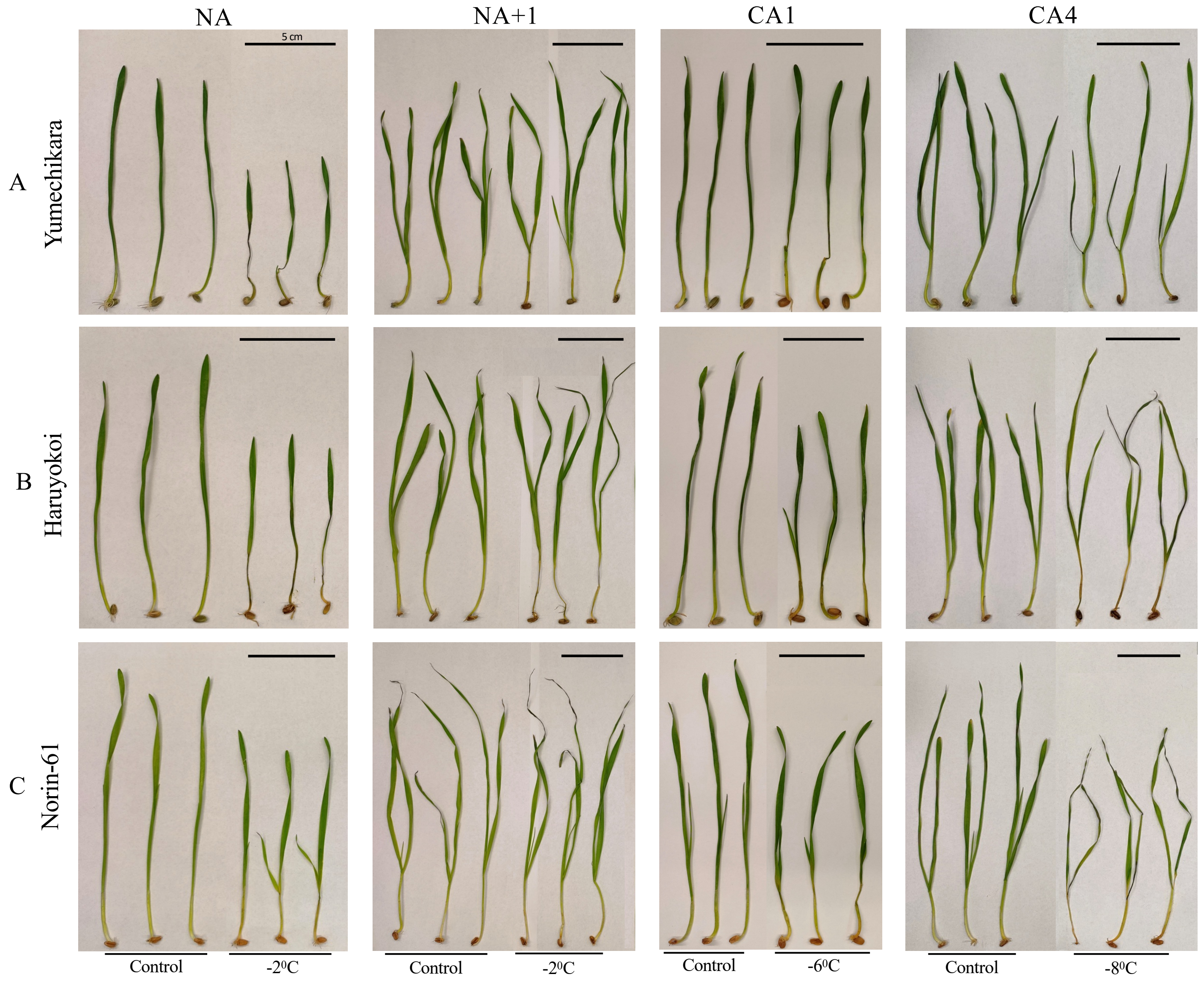

Supplementary Figure S3. Grow out tests of the three wheat cultivars. (A) Yumechikara, (B) Haruyokoi and (C) Norin-61. Each horizontal row presents the respective wheat cultivar and each column shows the acclimation stages (NA, NA+1, CA1 and CA4). Plants in test tubes were frozen and placed at room temperature for 72 hours before photographs were taken. Scale bars represent 5 cm.

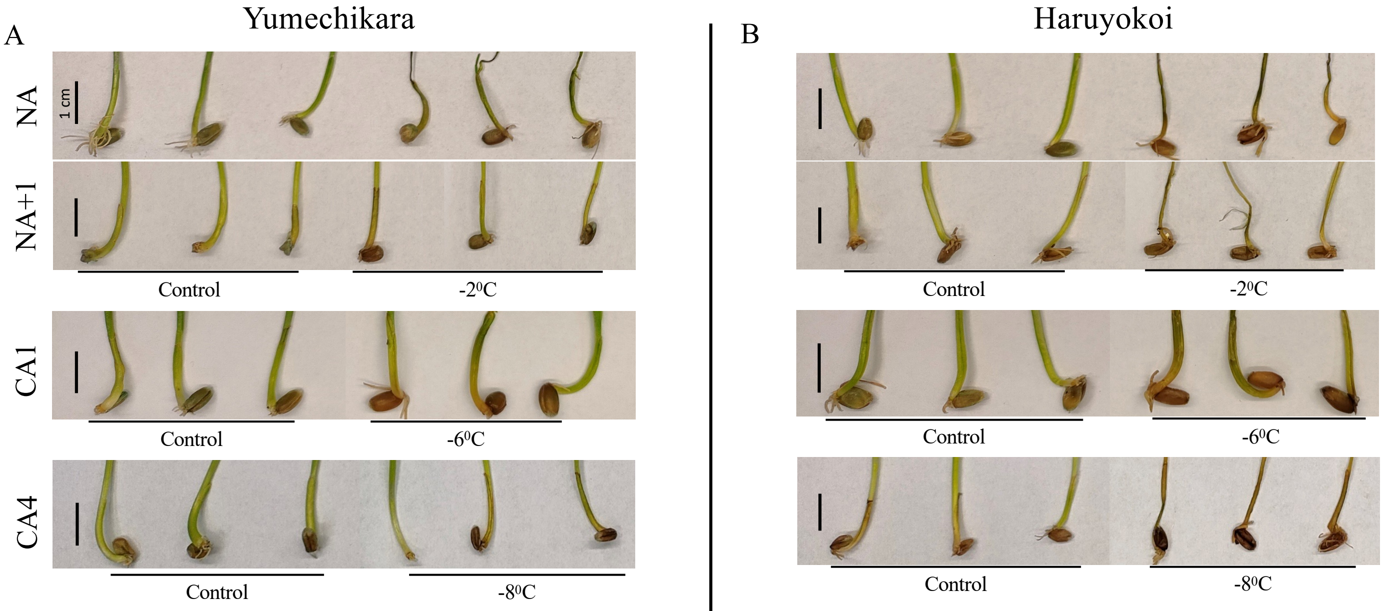

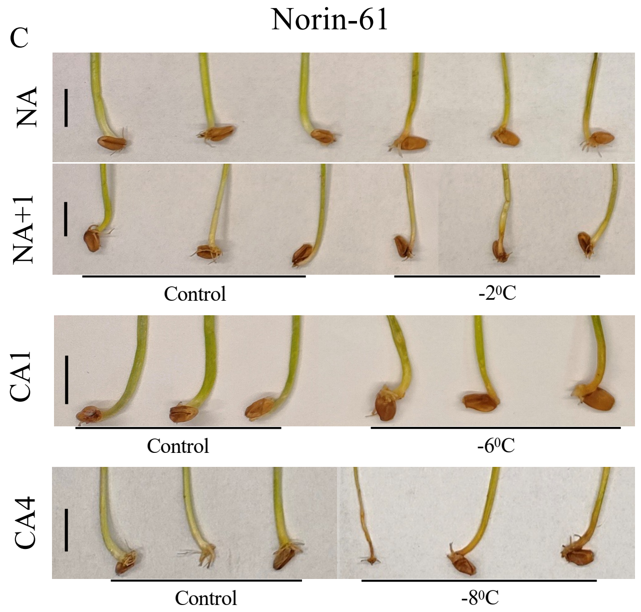

Supplementary Figure S4. Morphological changes in the crown parts after freeze-thawing in plants undergoing different acclimation stages (NA, NA+1, CA1, CA4) of (A) Yumechikara, (B) Haruyokoi and (C) Norin-61 wheat crowns. Plants in test tubes were frozen and placed at room temperature for 72 hours before photographs were taken. Scale bars represent 1 cm.

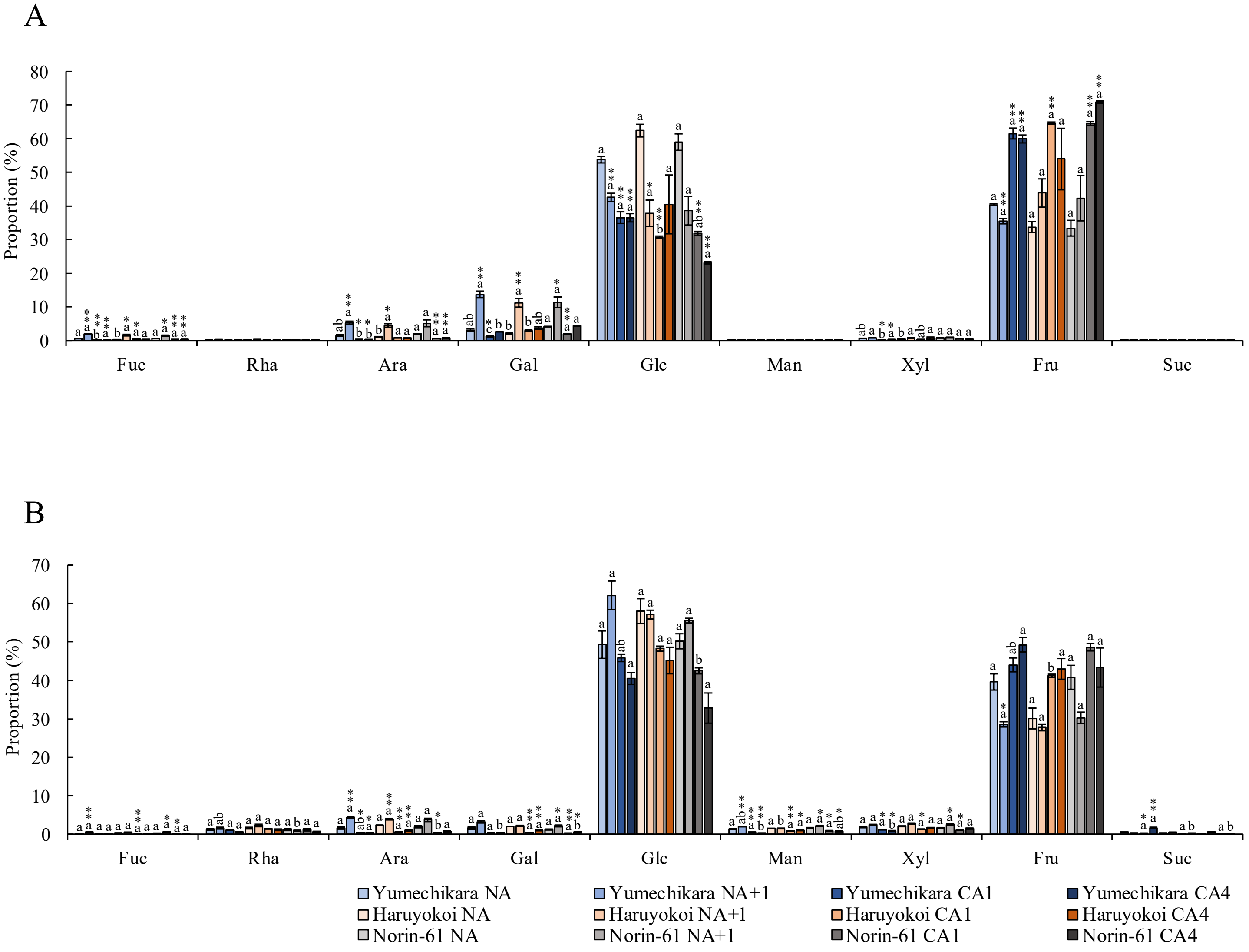

Supplementary Figure S5. Ratio of constituent sugars in soluble fraction derived from NA and CA samples. (A) Leaves (above ground part) and (B) Crown were fractionated to extract the soluble sugars. Monosaccharide compositional analysis was performed using HPAEC-PAD. Error bars represent ± SEM (n = 3). Significant differences according to the Tukey's HSD test among cultivars subjected to the same acclimation regime are indicated by different letters. Statistical differences with NA and other acclimation regimes were determined by Dunnett's test (*$p < 0.05$, **$p < 0.01$).

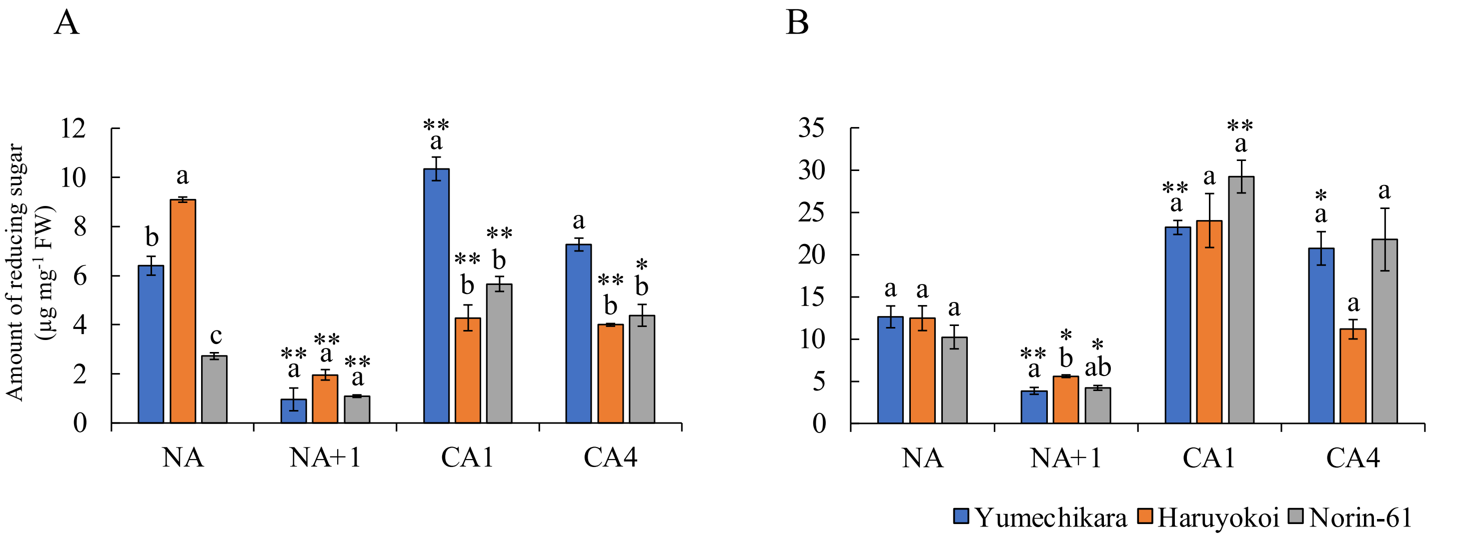

Supplementary Figure S6. Reducing sugar in wheat cultivars after different acclimation times. Amount of reducing sugar in (A) leaves and (B) the crown part of three wheat cultivars determined by the neocuproine test. Soluble fractions from the freshly harvested plants were used to estimate the reducing sugar. Error bars indicate ± SEM (n=3). Significant differences among cultivars obtained from the Tukey's HSD test applied within the same acclimation regimes are indicated by different letters. Statistical differences with NA and other acclimation points were determined using Dunnett's test (*p < 0.05, **p < 0.01).

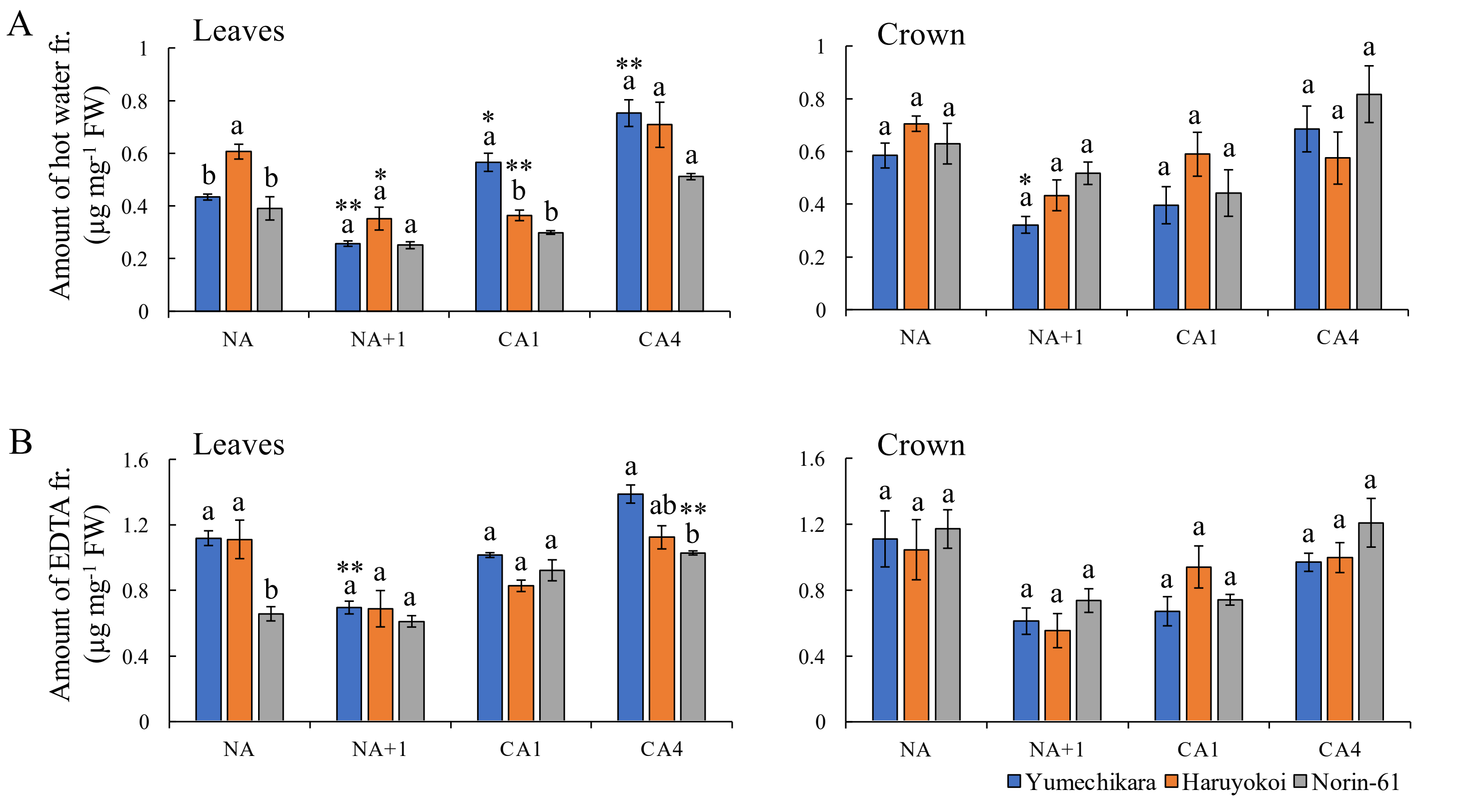

Supplementary Figure S7. Changes in total sugar content of cell wall polysaccharides at different acclimation stages in leaves and crown parts. (A) Hot water fraction and (B) EDTA fraction. The Y-axis indicates relative sugar amount in microgram per milligram of fresh weight and the X-axis represents the four acclimation stages. Error bars indicate ± SEM (n = 3). Significant differences among cultivars inferred from the Tukey's HSD test for each acclimation point are indicated by different letters. Statistical differences with NA and other acclimation points were determined with Dunnett's test (*p < 0.05, **p < 0.01).

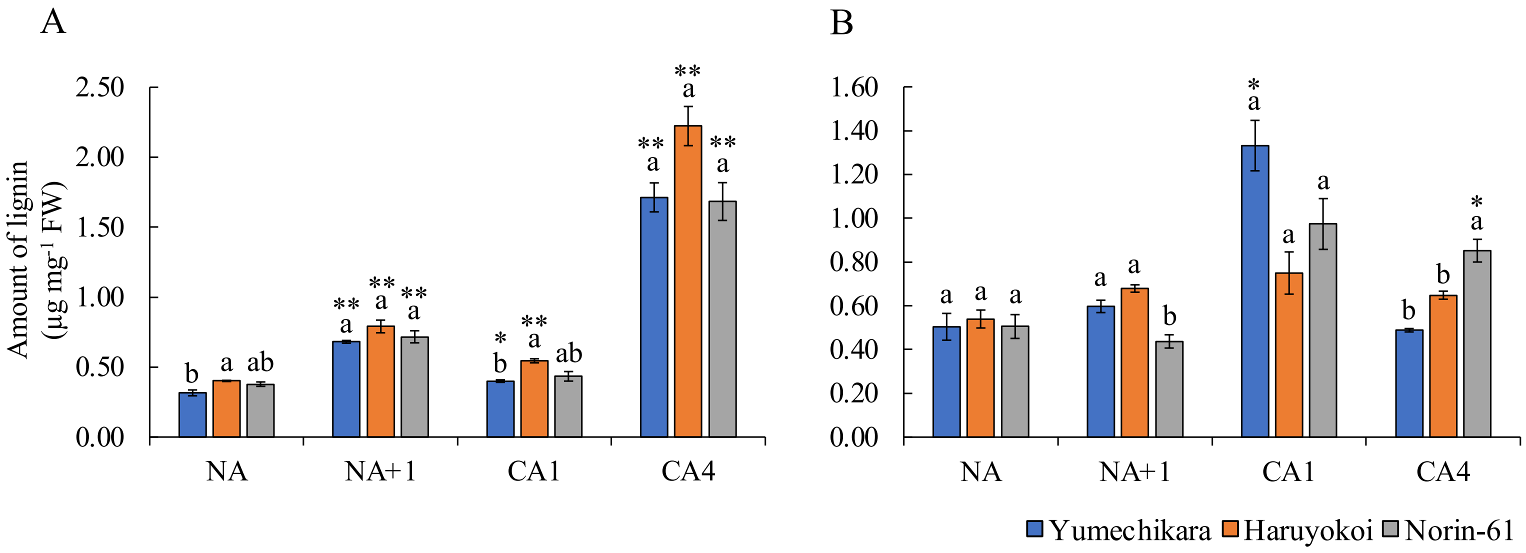

Supplementary Figure S8. Changes in lignin content of the three wheat cultivars during cold acclimation. (A) lignin content in leaves, and (B) lignin content in crown parts. The Y-axis denotes relative sugar amounts in microgram per milligram of fresh weight and the X-axis represents the four acclimation stages. Error bars indicate ± SEM (n = 3). Significant differences according to the Tukey's HSD test among cultivars within a given acclimation regime are indicated by different letters. Statistical differences with NA and other acclimation points were determined with Dunnett’s test (*p < 0.05, **p < 0.01).

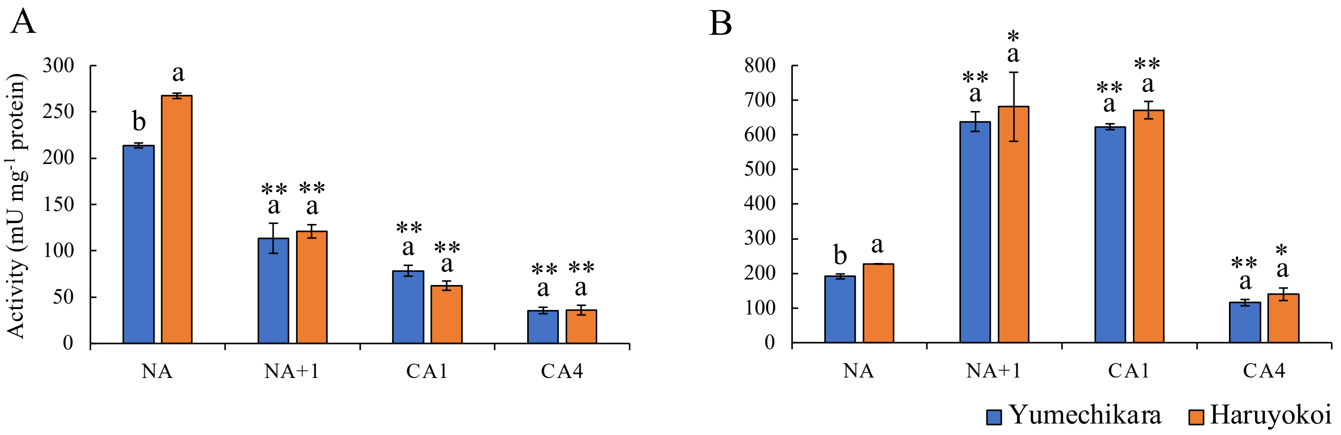

Supplementary Figure S9. Activity based on protein content in wheat cultivars for different acclimation times. (A) Invertase, and (B) amylase activity per milligram of protein. Freshly harvested plants were used to measure protein content in two wheat cultivars. Error bars indicate ± SEM (n = 3). Significant differences among cultivars subjected to the same acclimation regime are indicated by different letters (Tukey's HSD test). Statistical differences with NA and other acclimation procedures were determined with Dunnett’s test (*$p < 0.05$, **$p < 0.01$).

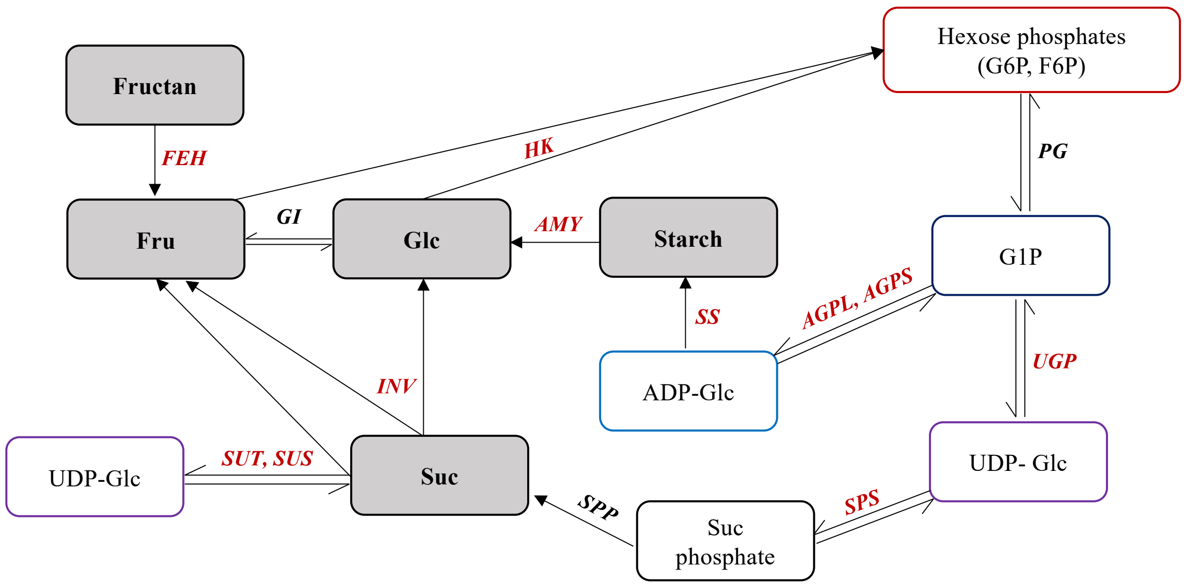

Supplementary Figure S10. Possible pathways of Glc and fru metabolism in Yumechikara and Haruyokoi. Red color indicates that expression of these enzymes was checked in this study. Abbreviations: INV, invertase; SUT, Suc transporter; SUS, Suc synthase; SPS, Suc phosphate synthase; FEH, fructan 6-exohydrolase; HK, hexokinase; UGP, UDP-Glc pyrophosphorylase; SS, starch synthase; AGPS, ADP-Glc pyrophosphorylase small subunit; AGPL, ADP-Glc pyrophosphorylase large subunit; AMY, amylase; PG, phosphoglucomutase; SPP, Suc phosphate phosphatase; GI, Glc isomerase; G6P, Glc-6-phosphate; F6P, Fru-6-phosphate; G1P, Glc-1-phosphate; UDP-Glc, uridine diphosphate Glc; ADP-Glc, adenosine diphosphate Glc.

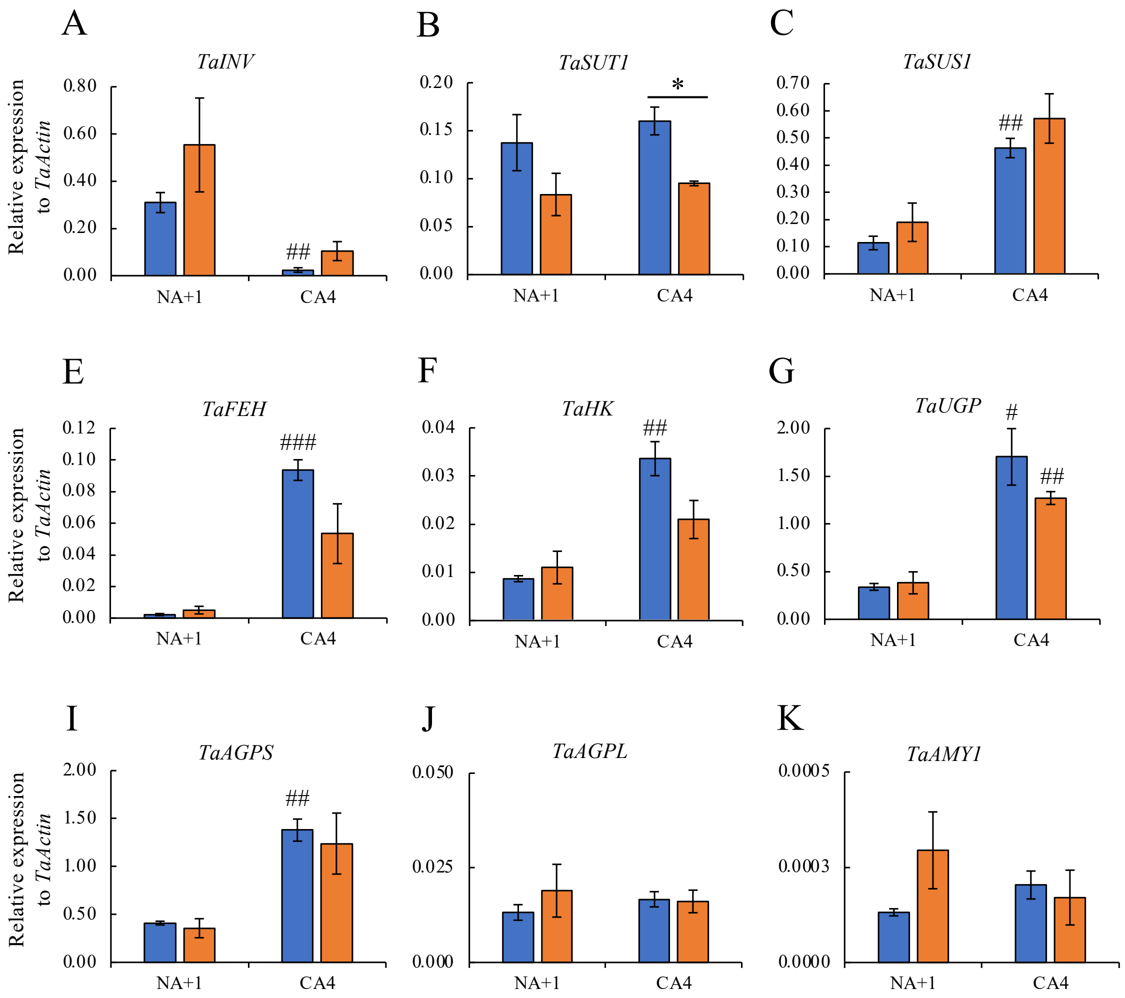

Supplementary Figure S11. Differential expression level of starch and Suc metabolism enzymes in Yumechikara and Haruyokoi under NA and CA. Expression pattern of (A) *TaINV*, (B) *TaSUT1*, (C) *TaSUS1*, (D) *TaSPS*, (E) *TaFEH*, (F) *TaHK*, (G) *TaUGP*, (H) *TaSS1*, (I) *TaAGPS*, (J) *TaAGPL*, (K) *TaAMY1*. Values represent the expression levels relative to that of *TaActin*. Error bars indicate ± SEM (n = 3). Significant differences (t-test) are indicated by asterisks among the bars and hash (#) symbols indicate comparison with NA+1 ($^{*/\#}p < 0.05$, $^{\#\#}p < 0.01$, $^{\#\#\#}p < 0.001$).

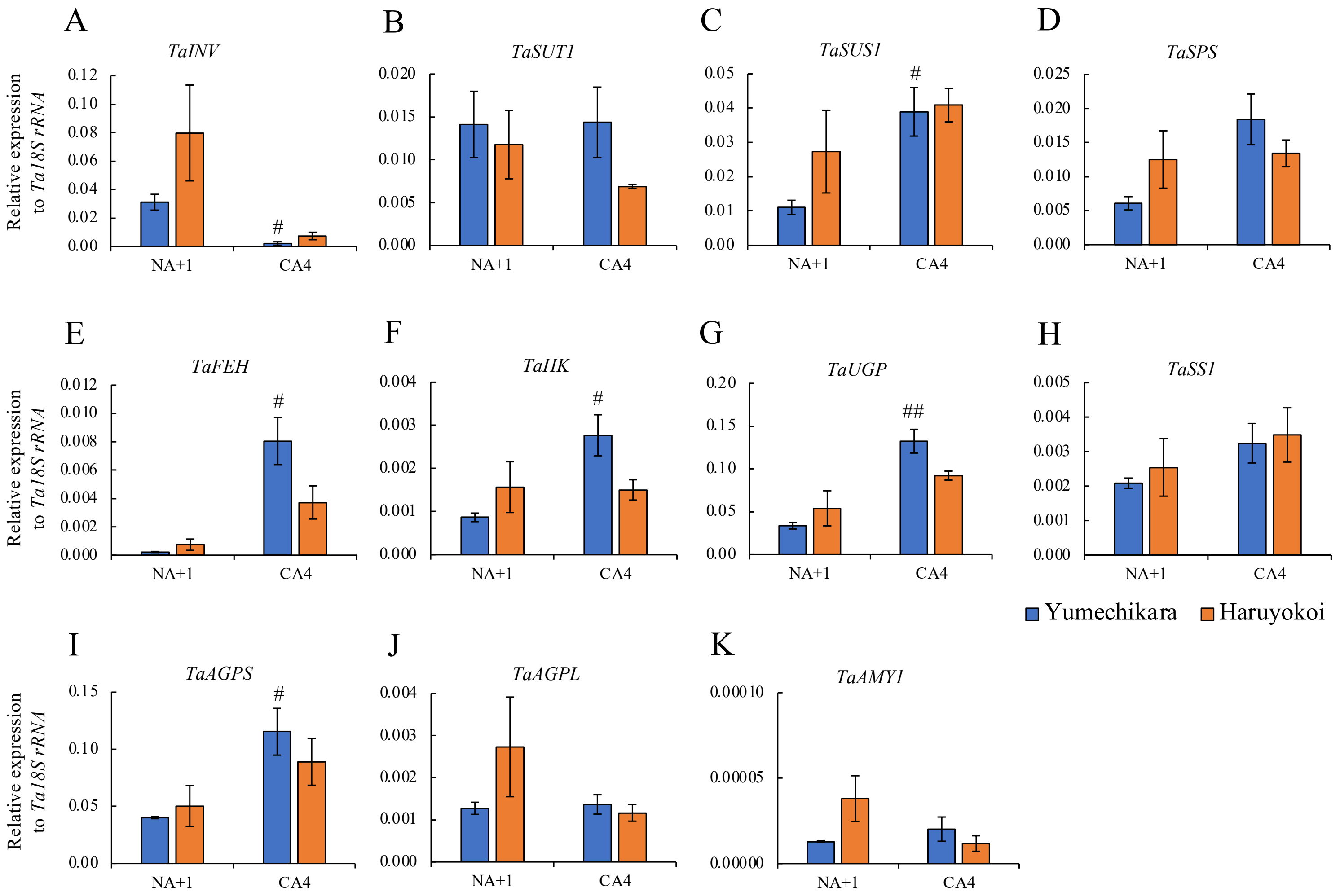

Supplementary Figure S12. Differential expression level of starch and Suc metabolism enzymes in Yumechikara and Haruyokoi under NA and CA. Expression pattern of (A) *TaINV*, (B) *TaSUT1*, (C) *TaSUS1*, (D) *TaSPS*, (E) *TaFEH*, (F) *TaHK*, (G) *TaUGP*, (H) *TaSS1*, (I) *TaAGPS*, (J) *TaAGPL*, (K) *TaAMY1*. Values represent the expression levels relative to that of *Ta18S rRNA*. Error bars indicate ± SEM (n = 3). Hash (#) symbols indicate significant differences (t-test) compared to NA+1 ($^{\#}p < 0.05$, $^{\#\#}p < 0.01$). No statistical significance was found between cultivars.

Supplementary Table S1. Features of the studied wheat cultivars

**Characteristics**	**Yumechikara**	**Haruyokoi**	**Norin-61**
Growth habit	winter	spring	winter
Plant height (cm)	85	90	92
Ear length (cm)	9.8	8.5	8.5
1000 kernel weight (g)	42	40	41
Days to maturity (App.)	170	130	160
Grain Yield (t/ha)	8.34	4.0	3.8

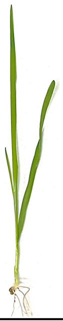
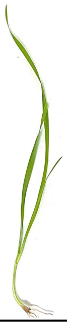
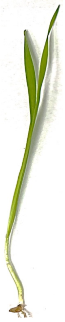

* Average value of the respective trait is calculated based on field trials
(Miyawaki et al., 2013; Tabiki et al., 2011; Tanio et al., 2005; Yanagisawa and Tabiki, 2002)

Supplementary Table S2. List of primers used for RT-qPCR.

Sl.	Gene	Forward primer	Reverse primer	Reference
1	*TaTUB*	CCCTGAGGTTTGATGGTGCT	TGGTGATCTCAGCAACGGAC	Dudziak et al. 2020
2	*TaActin*	CCTTAGTACCTTCCAACAGATGT	CCAGACAACTCGCAACTTAGA	Zeng et al. 2011
3	*Ta18S rRNA*	GTCAGAGGTGAAATTCTTGGATTTA	AGGGCAGGGACGTAATCAACG	Nemati et al. 2018
4	*TaINV*	GACGACTCCTACAACCAGAT	CTCCATCTCCATCCATCCAT	Bagherikia et al. 2018
5	*TaSUT1*	CGCATAGGCGTAACTAGGG	CACATACAGACACAACACATACA	
6	*TaSUS1*	GGACAGGAACAAGCCAATCATC	TCTTGCCGTACATCTCAACCAA	
7	*TaFEH*	GAGCACACGGCTATCTTCTTCA	CCACTACCGAATGGTCAATCAA	
8	*TaSPS*	ATCGTCACGCTCGCTCAA	AGTCATCTTCCTGCCAAAATTACA	Zeng et al. 2011
9	*TaUGP*	TTGAAGGTCTCTGGTGAT	CGTTGATGTCCTTGTTCT	
10	*TaHK*	AAACGTGCTGTCCAACAATATG	TCGTCTCCTTCAGTCTCAACAA	Wang et al. 2022
11	*TaSS1*	GCAAAAGGAGAGGAGGGTACA	ACGTATGGTCTTTCGTCATGC	Wang et al. 2014
12	*TaAGPS*	ACACACAACCTCGACACTTG	TGCCACCTTTTTCAGCAAGG	Gu et al. 2021
13	*TaAGPL*	GAACTACATGACTTTGGGTCTGAGA	CATCATCATCGCATTCTTGAGCT	Zhang et al. 2019
14	*TaAMY1*	TCGATGTGGGGCACCTCATTC	TCGTACTATGTGGACAACATGAC	Barrero et al. 2013

References

Bagherikia S, Pahlevani M, Yamchi A, Zaynalinezhad K, Mostafaie A (2018) Transcript profiling of genes encoding fructan and sucrose metabolism in wheat under terminal drought stress. *J Plant Growth Regul* 38: 148-163 https://doi.org/10.1007/s00344-018-9822-y

Barrero JM, Mrva K, Talbot MJ, White RG, Taylor J, Gubler F, Mares DJ (2013) Genetic, hormonal, and physiological analysis of late maturity α-amylase in wheat. *Plant Physiol* 161: 1265-1277 https://doi.org/10.1104/pp.112.209502

Dudziak K, Sozoniuk M, Szczerba H, Kuzdraliński A, Kowalczyk K, Börner A, Nowak M (2020) Identification of stable reference genes for qPCR studies in common wheat (*Triticum aestivum* L.) seedlings under short-term drought stress. *Plant Methods* 16: 58 https://doi.org/10.1186/s13007-020-00601-9

Gu Y, Han S, Chen L, Mu J, Duan L, Li Y, Yan Y, Li X (2021) Expression and regulation of genes involved in the reserve starch biosynthesis pathway in hexaploid wheat (*Triticum aestivum* L.). *Crop J* 9: 440-455 https://doi.org/10.1016/j.cj.2020.08.002

Miyawaki T, Matsumoto J, Ogawa T, Yoshimura Y, Iwai M (2013) Growth and milling characteristics of wheat cultivars 'Kitahonami' and 'Yumechikara' in Hyogo prefecture. *235th Crop Science Society of Japan Lecture Meeting* 2013: 70 https://doi.org/10.14829/jcsproc.235.0_70

Nemati F, Ghanati F, Ahmadi Gavlighi H, Sharifi M (2018) Comparison of sucrose metabolism in wheat seedlings during drought stress and subsequent recovery. *Biol Plant* 62: 595-599 https://doi.org/10.1007/s10535-018-0792-5

Tabiki T, Nishio Z, Ito M, Yamauchi H, Takata K, Kuwabara T, Iriki N, Tanio M, Ikeda T, Funatsuki W (2011) A new extra- strong hard red winter wheat variety: 'Yumechikara'. *Research Bulletin of the NARO Hokkaido Agricultural Research Center* 195: 1-12

Tanio M, Kato K, Ishikawa N, Tamura Y, Sato M, Takagi H, Matsuoka M (2005) Genetic analysis of photoperiod response in wheat and its relation with the earliness of heading in the southwestern part of Japan. *Breed Sci* 55: 327-334 https://doi.org/10.1270/jsbbs.55.327

Wang W, Wang X, Lv Z, Khanzada A, Huang M, Cai J, Zhou Q, Huo Z, Jiang D (2022) Effects of cold and salicylic acid priming on free proline and sucrose accumulation in winter wheat under freezing stress. *J Plant Growth Regul.* 41: 2171-2184 https://doi.org/10.1007/s00344-021-10412-4

Wang Z, Li W, Qi J, Shi P, Yin Y (2014) Starch accumulation, activities of key enzyme and gene expression in starch synthesis of wheat endosperm with different starch contents. *J Food Sci Technol* 51: 419-429 https://doi.org/10.1007/s13197-011-0520-z

Yanagisawa A, Tabiki T (2002) A new spring wheat variety "Haruyokoi". *Hokkaido agricultural survey report* 82: 113-116. URL: https://www.hro.or.jp/list/agricultural/center/kankoubutsu/syuhou/82a/82-14.pdf (Accessed December 15, 2023)

Zeng Y, Yu J, Cang J, Liu L, Mu Y, Wang J, Zhang D (2011) Detection of sugar sccumulation and expression levels of correlative key enzymes in winter wheat (*Triticum aestivum*) at low temperatures. *Biosci Biotechnol Biochem* 75: 681- 687 https://doi.org/10.1271/bbb.100813

Zhang S, Guo H, Irshad A, Xie Y, Zhao L, Xiong H, Gu J, Zhao S, Ding Y, Liu L (2019) The synergistic effects of *TaAGP.L-B1* and *TaSSIVb-D* mutations in wheat lead to alterations of gene expression patterns and starch content in grain development. *PLoS One* 14: e0223783 https://doi.org/10.1371/journal.pone.0223783
